# Association of Vaginal Estradiol Tablet With Serum Estrogen Levels in Women Who Are Postmenopausal: Secondary Analysis of a Randomized Clinical Trial

**DOI:** 10.1001/jamanetworkopen.2022.41743

**Published:** 2022-11-14

**Authors:** Caroline M. Mitchell, Joseph C. Larson, Carolyn J. Crandall, Shalender Bhasin, Andrea Z. LaCroix, Kristine E. Ensrud, Katherine A. Guthrie, Susan D. Reed

**Affiliations:** 1 Vincent Center for Reproductive Biology, Massachusetts General Hospital, Boston; 2 Public Health Sciences Division, Fred Hutchinson Cancer Research Center, Seattle, Washington; 3 Department of Medicine, David Geffen School of Medicine at University of California, Los Angeles; 4 Research Program in Men’s Health: Aging and Metabolism, Boston Claude D. Pepper Older Americans Center, Brigham and Women’s Hospital, Harvard Medical School, Boston, Massachusetts; 5 Herbert Wertheim School of Public Health, University of California, San Diego; 6 Departments of Medicine and Epidemiology and Community Health, University of Minnesota, Minneapolis; 7 Center for Care Delivery and Outcomes Research, Minneapolis VA Health Care System, Minneapolis, Minnesota; 8 Department of Obstetrics and Gynecology, University of Washington, Seattle

## Abstract

**Importance:**

Half of women who are postmenopausal have genitourinary discomfort after menopause. Recommended therapies include low-dose vaginal estrogen. Individuals with a history of breast cancer or venous thromboembolism may have concerns about the safety of this intervention.

**Objective:**

To compare serum estrogen concentrations with the use of vaginal estrogen, 10 μg, tablet vs placebo in women who are postmenopausal.

**Design, Setting, and Participants:**

This is a secondary, post hoc analysis of data from a randomized clinical trial of treatment for moderate to severe genitourinary syndrome in women who are postmenopausal. The study was conducted at Kaiser Permanente Washington Health Research Institute and the University of Minnesota from April 11, 2016, to April 23, 2017. Measurements and data analysis were performed from November 3, 2020, to September 23, 2022.

**Interventions:**

Participants were randomly assigned to vaginal estradiol tablet (10 μg/d for 2 weeks and then twice weekly) plus placebo gel (3 times weekly) or dual placebo for 12 weeks.

**Main Outcomes and Measures:**

In this post hoc analysis, baseline and week 12 serum estradiol, estrone, and sex hormone–binding globulin (SHBG) concentrations were measured by a chemiluminescent assay. Week 12 values of the 3 analytes were compared by baseline participant characteristics. Linear models compared week 12 estradiol concentrations between treatment groups, adjusted for baseline characteristics.

**Results:**

A total of 174 women, mean (SD) age 61 (4) years, were included. Those in the estrogen group (n = 88) were more likely to have higher geometric mean (SD) week 12 serum estradiol concentrations (4.3 [2.2 pg/mL]) than those in the placebo group (n = 86) (3.5 [2.1] pg/mL) (*P* = .01). Adjusted for pretreatment hormone concentrations, age, clinical site, and body mass index, assignment to the estrogen vs placebo treatment group was significantly associated with higher week 12 estradiol concentrations (23.8% difference; 95% CI, 6.9%-43.3%). Most (121 of 174 [69.5%]) participants had enrollment serum estradiol concentrations higher than 2.7 pg/mL. Of women starting treatment at estradiol levels lower than or equal to 2.7 pg/mL, 38.1% (8 of 21) in the estrogen group and 34.4% (11 of 32) in the placebo group had estradiol concentrations higher than 2.7 pg/mL after 12 weeks of study participation (*P* = .78). Treatment assignment was not associated with week 12 estrone or SHBG concentrations.

**Conclusions and Relevance:**

In this secondary analysis of a randomized clinical trial, a significant, although small, increase in serum estradiol levels was noted after 12 weeks of vaginal estrogen administration. The clinical relevance of this small increase is uncertain.

**Trial Registration:**

ClinicalTrials.gov Identifier: NCT02516202

## Key Points

**Question:**

Is low-dose vaginal estradiol treatment associated with increases in serum concentrations of estradiol, estrone, or sex hormone–binding globulin (SHBG)?

**Findings:**

In this secondary analysis of randomized clinical trial data on 174 women who are postmenopausal, 12 weeks of vaginal estradiol, 10 µg, tablet use was associated with a higher median serum estradiol concentration, but not estrone or SHBG concentration, vs placebo. The proportions of participants whose serum estradiol concentration shifted from a lower (≤2.7 pg/mL) to a higher (>2.7 pg/mL) risk category varied little by treatment group.

**Meaning:**

Low-dose vaginal estradiol use was associated with a small increase in serum estradiol concentrations of uncertain clinical significance.

## Teaser

This secondary analysis of a randomized clinical trial examines serum estrogen concentrations following treatment with a low-dose vaginal estradiol tablet in women who are postmenopausal.

## Introduction

With heightened caution around the use of systemic estrogens after publication of results from the Women’s Health Initiative,^1^ many health care professionals and patients are also concerned about the risks of low-dose vaginal estrogen formulations to treat genitourinary symptoms. The same warning labels and reported adverse effects are listed for both oral and vaginal formulations, which deters some people from opting to use vaginal estrogen preparations. However, systemic absorption with the use of vaginal estrogen has been shown to be very low.^2,3,4^ The systemic absorption of estrogen when administered vaginally is of particular concern for women with a history of breast cancer and individuals with a history of cardiovascular disease or deep venous thrombosis.

Current guidance from the American College of Obstetricians and Gynecologists considers low-dose vaginal estrogen a reasonable treatment choice for women with a history of estrogen-dependent breast cancer.^5^ Observational studies, such as the Women’s Health Initiative and the Nurses’ Health study, found no association between vaginal estrogen use and risks of cardiovascular disease or cancer.^6,7^ In addition, vaginal estrogen use was not associated with a risk of breast cancer recurrence in multiple studies of individuals with a history of breast cancer.^8,9,10^ However, to our knowledge, no randomized clinical trials have been conducted to assess the risk for breast cancer or other adverse outcomes with use of vaginal estrogen preparations.

There are very few data to help define a safe systemic estradiol concentration after menopause. Serum estrogen levels are associated with individual participant characteristics, including body mass index (BMI).^11^ A systematic review found that common vaginal estrogen treatment regimens are associated with posttreatment mean estradiol concentrations ranging from 8.6 to 30 pg/mL (to convert to picomoles per liter, multiply by 3.671), depending on the type of estrogen product and type of assay used to measure the hormone.^3^ A meta-analysis of breast cancer risk with endogenous hormone concentrations^12^ used data from 9 studies with median serum estradiol levels ranging from 5.9 to 36.5 pg/mL and suggested greater risk for breast cancer with higher serum estradiol or estrone levels. In a randomized clinical trial of 7290 women with postmenopausal osteoporosis, those randomly assigned to placebo with baseline serum estradiol concentrations higher than 2.7 pg/mL had a significantly greater risk (6.8-fold higher) for breast cancer during 4 years compared with those with baseline concentrations lower than or equal to 2.72 pg/mL.^13^ Also, randomization to raloxifene treatment significantly reduced breast cancer risk compared with placebo, but only in the subset of women with baseline estradiol levels higher than 2.72 pg/mL. These results suggest that concentrations above this threshold may be associated with an increased risk for breast cancer.

We hypothesized that use of vaginal estrogen in women with bothersome vaginal symptoms would not lead to a significant increase in serum estradiol concentrations at 12 weeks. To test this hypothesis, we compared change in serum estradiol, estrone, and sex hormone–binding globulin (SHBG) concentrations during 12 weeks of treatment in the active vs placebo groups of a randomized clinical trial of a vaginal estradiol tablet for treatment of postmenopausal vaginal discomfort. We included SHBG because lower levels have independently been associated with cardiovascular risk^14,15^ and because oral estrogen treatment has been shown to increase SHBG levels.^16^ Because of the high level of imprecision and nonspecificity of estradiol and estrone immunoassays in the low range prevalent among women during menopause, we measured these hormones using a highly sensitive liquid chromatography with tandem mass spectrometry assay, certified by the Centers for Disease Control and Prevention (CDC) Hormone Standardization (HoSt) Program.^17^

## Methods

### Study Design

The MsFLASH Vaginal Health Trial was a randomized, double-blind, placebo-controlled, 12-week clinical trial conducted between April 11, 2016, and April 23, 2017, at 2 centers: Kaiser Permanente Washington Health Research Institute, Seattle, Washington, and University of Minnesota, Minneapolis.^18^ Participants were women aged 45 to 70 years, 2 or more years since last menses, who reported 1 or more moderate to severe symptoms of vulvovaginal itching, pain, dryness, or irritation experienced at least weekly within the past 30 days or pain with penetration at least once monthly. Women were randomly assigned 1:1:1 to the 3 treatment groups: estradiol, 10 µg, vaginal tablet plus placebo vaginal gel (estrogen group); placebo tablet plus vaginal moisturizing gel (moisturizer group); and placebo tablet plus placebo gel (placebo group). Women were instructed to insert the vaginal tablet daily for 2 weeks and then twice weekly for the remaining 10 weeks, and the vaginal gel every 3 days throughout the 12-week trial. The study was approved by institutional review boards at participating institutions, and participants provided written informed consent. Participants consented to use of samples to study postmenopausal health in the original informed consent. Evaluation of estrogen/hormone levels was part of the original analysis plan. Detailed methods were previously described according to the Consolidated Standards of Reporting Trials (CONSORT) reporting guideline, and we have included all information required by the CONSORT checklist herein (Trial Protocol in Supplement 1; eFigure 1 in Supplement 2).^18^

At enrollment, participants completed questionnaires about the most bothersome vaginal symptom severity, whether they had been sexually active in the past month, menopause quality of life,^19^ depression (9-item Patient Health Questionnaire; we eliminated 1 item [suicidality]),^20^ anxiety (7-item Generalized Anxiety Disorder),^21^ and insomnia (Insomnia Severity Index)^22^ at each visit.^18^ Vaginal samples were collected for pH measurement and Vaginal Maturation Index. Body mass index was calculated as weight in kilograms divided by height in meters squared. Race and ethnicity were self-identified. We included race and ethnicity as a variable because of reported racial and ethnic differences in the experience of menopause.^23^ Participants had blood samples obtained at enrollment and after 12 weeks of study intervention. In this analysis, we compared serum measurements of estradiol, estrone, and SHBG concentrations in the estrogen vs placebo treatment groups.

### Measurement of Serum Analytes

Serum total estradiol and total estrone concentrations were measured in 2020 by the Brigham Research Assay Core Laboratory using a liquid chromatography with tandem mass spectrometry (LC-MS/MS) method certified by the HoSt Program.^24,25^ For the estradiol and estrone assays, the lower limit of quantitation was 1 pg/mL (linear range, 1-500 pg/mL); the intraassay coefficient of variation was less than 5%, and the interassay coefficient of variation was less than 12%. For the LC-MS/MS estradiol assay, the mean bias for quality control specimens provided by the HoSt Program was 0.81 pg/mL for estradiol concentrations lower than or equal to 20 mg/mL and 1.9% for specimens with estradiol concentrations higher than 20 pg/mL. The imprecision was 4.6% in HoSt Program quality control pools with concentrations ranging from 2.6 to 24.2 pg/mL, 3.8% in the concentration range of 27.7 to 39.3 pg/mL, and 3.7% in the concentration range of 39.4 to 230.0 pg/mL.

### Statistical Analysis

We compared week 12 values of the 3 analytes according to categories of participant characteristics at enrollment and treatment assignment via analysis of variance (*F* tests). Linear regression models (Wald statistics) were used to evaluate the treatment assignment in week 12 analyte values. Model 1 adjusted for continuous baseline hormone concentration; model 2 additionally adjusted for clinical center, age (continuous), and BMI (continuous). We also compared baseline characteristics between people with week 12 estradiol levels lower than or equal to 2.7 pg/mL vs higher than 2.7 pg/mL. As sensitivity analyses, these models were also performed in the subset of participants who reported at least 80% adherence to treatment assignment.

Subgroup analyses were performed to evaluate whether the association between treatment group and week 12 serum estradiol levels varied according to levels of baseline estradiol, BMI, and years since menopause. A linear model of week 12 estradiol levels was fit for each baseline factor as a function of treatment group, the baseline factor, their interaction, and the adjustment factors from model 2 as appropriate.

Analyses were conducted using SAS, version 9.4 (SAS Institute Inc) with 2-sided values of *P* = .05 considered statistically significant. Statistical analyses were completed between June 21, 2021, and September 23, 2022.

## Results

This secondary analysis included 174 participants from the MsFLASH Vaginal Health Trial who were assigned to the vaginal estrogen group (n = 88) or the placebo group (n = 86) and had blood samples available from both enrollment and week 12 visits. Racial and ethnic groups comprised African American (8 [4.6%]), White (152 [87.4%]), and other or unknown (14 [8.0%]) individuals. The mean (SD) age was 61 (4) years, and the mean time from menopause was 11 (7) years (Table 1).

**Table 1.** Associations Between Baseline Participant Characteristics and Week 12 Estradiol Concentration

Characteristic	No. (%)^a^	Week 12 estradiol, geometric mean (SD), pg/mL	*P* value^b^
Treatment group			
Vaginal estrogen	88 (50.6)	4.3 (2.2)	.01
Placebo	86 (49.4)	3.5 (2.1)	
Study pill adherence, %			
<80	22 (12.6)	3.4 (2.0)	.25
≥80	152 (87.4)	4.0 (2.2)	
Age, y			
<60	72 (41.3)	3.7 (2.2)	.40
≥60	102 (58.7)	4.0 (2.3)	
Time since menopause, y			
<5	25 (14.4)	4.3 (3.0)	.36
≥5	147 (84.5)	3.8 (2.1)	
BMI			
<25	82 (47.1)	3.2 (1.6)	<.001
≥25	92 (52.9)	4.6 (2.6)	
Estradiol concentration			
<Median (3.5 pg/mL)	87 (50.0)	2.8 (1.4)	<.001
≥Median	87 (50.0)	5.3 (2.4)	
Estrone concentration			
<Median (10.5 pg/mL)	87 (50.0)	3.4 (2.0)	.003
≥Median	87 (50.0)	4.4 (2.3)	
SHBG concentration			
<Median (6.67 μg/mL)	87 (50.0)	4.2 (2.5)	.05
≥Median	87 (50.0)	3.6 (1.9)	
Race and ethnicity			
African American	8 (4.6)	6.3 (2.4)	.05
White	152 (87.4)	3.8 (2.2)	
Other or unknown^c^	14 (8.0)	4.0 (2.1)	
Sexually active			
Yes	143 (82.2)	3.8 (2.2)	.32
No	30 (17.2)	4.3 (2.4)	
Most bothersome symptom^d^			
Pain with vaginal penetration	97 (57.1)	3.7 (2.2)	.87
Vaginal dryness	38 (21.8)	4.2 (2.3)	
Vulvar or vaginal itching	15 (8.6)	3.7 (2.2)	
Vulvar or vaginal irritation	13 (7.5)	4.1 (2.5)	
Vulvar or vaginal soreness	7 (4.0)	4.1 (1.4)	
Vaginal pH			
≤5	22 (12.6)	5.0 (2.3)	.03
>5	151 (86.8)	3.7 (2.2)	
Vaginal Maturation Index, % superficial cells			
≤5	143 (82.2)	3.8 (2.3)	.15
>5	11 (6.3)	5.0 (2.4)	
MENQOL score, total^e^			
<Median (<3.16)	83 (47.7)	3.4 (1.7)	.003
≥Median	84 (48.3)	4.4 (2.6)	
PHQ-9 depression score^f^			
No symptoms (<5)	121 (69.5)	3.6 (1.9)	.01
Mild or worse symptoms	52 (29.9)	4.6 (3.0)	
GAD-7 anxiety score^g^			
No symptoms (<5)	108 (62.1)	3.9 (2.1)	.73
Mild or worse symptoms	65 (37.4)	4.0 (2.4)	
ISI score^h^			
No symptoms (<8)	92 (52.8)	3.7 (1.9)	.19
Mild or worse symptoms	81 (46.6)	4.1 (2.6)	

Abbreviations: BMI, body mass index (calculated as weight in kilograms divided by height in meters squared); GAD-7, 7-item Generalized Anxiety Disorder; ISI, Insomnia Severity Index; MENQOL, Menopause Quality of Life; PHQ-9, 9-item Patient Health Questionnaire; SHBG, sex hormone–binding globulin.

SI conversion: to convert estradiol to picomoles per liter, multiply by 3.671; estrone to picomoles per liter, multiply by 3.698; SHBG to nanomoles per liter, multiply by 8.896.

^a^ Sums may differ and percentages may not add up to 100% due to missing characteristic data.

^b^ *P* values determined from an analysis of variance model of log-transformed week 12 estradiol levels by category of participant characteristic.

^c^ Other includes American Indian or Alaska Native, Asian or Pacific Islander, Hispanic, or unknown. Groups were combined due to small numbers. *P* value compares race as White vs African American, American Indian or Alaska Native, Asian or Pacific Islander, Hispanic, or unknown.

^d^ *P* value compares pain with vaginal penetration vs all other symptoms.

^e^ A higher MENQOL score is considered worse quality of life (range, 1-8).^15^

^f^ The PHQ-9 measures depression. A PHQ-9 score less than 5 indicates no depression (range, 0-20); we eliminated 1 item (suicidality).^16^

^g^ The GAD-7 scale measures anxiety. A GAD-7 score less than 5 indicates no anxiety (range, 0-17).^18^

^h^ The ISI measures insomnia. An ISI score less than 8 indicates no insomnia (range, 0-27).^19^

Participants assigned to the vaginal estrogen group had higher serum estradiol levels at week 12 compared with the placebo group (geometric mean [SD], 4.3 [2.2] vs 3.5 [2.1] pg/mL) (Table 1; Figure). Higher estradiol concentrations at week 12 were associated with baseline characteristics: higher BMI, higher estradiol and estrone levels at enrollment, lower SHBG level at enrollment, and higher (ie, worse) menopause quality of life scores. In addition, higher week 12 estradiol levels were significantly associated with lower vaginal pH at enrollment. Higher concentrations of serum estrone or serum SHBG at 12 weeks were also associated with higher BMI and serum estrone or SHBG levels at enrollment, but not with treatment assignment (eTable 1, eTable 2, eFigure 2, and eFigure 3 in Supplement 2).

**Figure.**
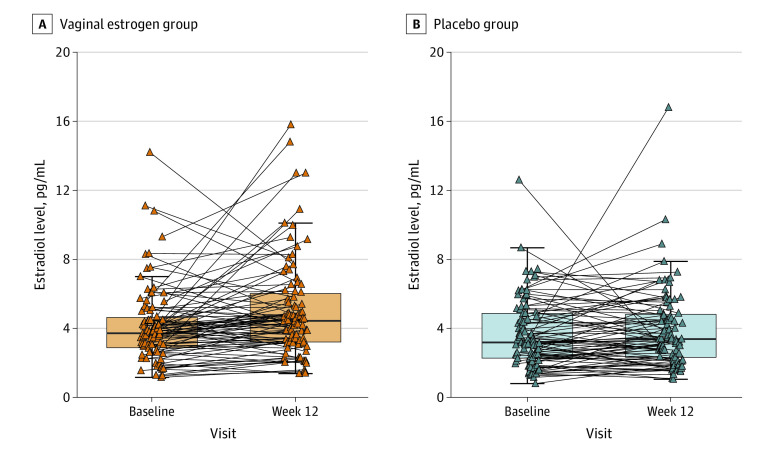
Serum Concentrations of Estradiol at Enrollment and 12 Weeks in Participants of the MsFLASH Vaginal Health Randomized Clinical Trial The box plots represent the median and IQR of values at enrollment and week 12. Each triangle represents a measurement from an individual participant, and the lines connect the pretreatment and week 12 values for each participant assigned to the vaginal estradiol, 10-µg, tablet plus placebo gel (n = 88) group (A) or the dual-placebo group (n = 86) (B). This summary does not include 2 participants who received placebo with unusually large estradiol levels (baseline: 40.3 pg/mL; week 12, 3.4 pg/mL and baseline, 7.4 pg/mL; week 12, 52.0 pg/mL).

When adjusted for pretreatment estradiol concentrations, assignment to the vaginal estrogen vs placebo treatment group was significantly associated with a higher week 12 estradiol concentration (24.5% difference; 95% CI, 6.6%-45.5%). Adjusting for age, clinical site, and BMI did not substantially alter this estimate (23.8% difference; 95% CI, 6.9%-43.3%) (Table 2). Model results were similar when limited to a subset of treatment-adherent participants. As expected from the univariate analysis, multivariable regression revealed no associations between treatment group week 12 serum estrone or SHBG concentrations (Table 2).

**Table 2.** Associations Between Treatment Group and Week 12 Hormone Concentrations

Outcome	Model^a^	Vaginal estrogen vs placebo, % difference (95% CI)^b^	*P* value
Estradiol	1	24.5 (6.6 to 45.5)	.006
	2	23.8 (6.9 to 43.3)	.004
Estrone	1	−3.3 (−13.7 to 8.5)	.57
	2	−2.5 (−13.0 to 9.3)	.66
SHBG	1	6.1 (−7.5 to 21.7)	.40
	2	7.7 (−5.6 to 22.9)	.27

Abbreviation: SHBG, sex hormone–binding globulin.

SI conversion: to convert estradiol to picomoles per liter, multiply by 3.671; estrone to picomoles per liter, multiply by 3.698; SHBG to nanomoles per liter, multiply by 8.896.

^a^ Estimates, 95% CIs, and *P* values for treatment group effect calculated from a linear model of log-transformed week 12 hormone concentration as a function of intervention arm with model 1, adjusted for relevant baseline hormone concentration (continuous), and model 2, additionally adjusted for clinical center (Kaiser Permanente Washington Health Research Institute, University of Minnesota), age (continuous), and body mass index (continuous).

^b^ Percent difference derived by exponentiating model estimates.

To better evaluate the potential clinical relevance of this association, we compared characteristics between women with 12-week estradiol concentrations higher than vs lower than or equal to the value (2.7 pg/mL) previously shown to be associated with an increased risk for breast cancer. A 12-week serum estradiol concentration higher than 2.7 pg/mL was associated with higher BMI, higher baseline serum concentrations of estradiol and estrone, and lower vaginal pH at enrollment (Table 3). Of note, 121 of 174 participants (69.5%) entered the trial with serum estradiol levels above this threshold. Of those starting at or below the threshold of 2.7 pg/mL, 38.1% (8 of 21) in the vaginal estrogen group and 34.4% (11 of 32) in the placebo group had estradiol concentrations higher than 2.7 pg/mL after 12 weeks of study participation (*P* = .78). Of participants with a starting concentration above 2.7 pg/mL, a similar proportion in each group (3 of 21 [4.3.%] vs 4 of 54 [7.4%]; *P* = .49) had a week 12 concentration below that threshold.

**Table 3.** Association Between Baseline Participant Characteristics and Week 12 Estradiol Concentration Higher Than 2.7 pg/mL vs Lower Than or Equal to 2.7 pg/mL

Characteristic	Week 12 estradiol, No. (%)^a^		*P* value^b^	
	≤2.7 pg/mL (n = 41)	>2.7 pg/mL (n = 133)		
Treatment group				
Vaginal estrogen	16 (39.0)	72 (54.1)	.09	
Placebo	25 (61.1)	61 (45.9)		
Study pill adherence, %				
<80	7 (17.1)	15 (11.3)	.33	
≥80	34 (82.9)	118 (88.7)		
Age, y				
<60	17 (41.5)	55 (41.4)	.99	
≥60	24 (58.5)	78 (58.6)		
Time since menopause, y				
<5	4 (9.8)	21 (15.8)	.36	
≥5	36 (87.8)	111 (83.5)		
BMI				
<25	26 (63.4)	56 (42.1)	.02	
≥25	15 (3.7)	77 (57.9)		
Estradiol concentration				
<Median (3.5 pg/mL)	39 (95.0)	48 (36.1)	<.001	
≥Median	2 (5.0)	85 (63.9)		
Estrone concentration				
<Median (10.5 pg/mL)	29 (70.7)	58 (43.6)	.003	
≥Median	12 (29.3)	75 (56.4)		
SHBG concentration				
<Median (6.67 μg/mL)	17 (41.5)	70 (52.6)	.21	
≥Median	24 (58.5)	63 (47.4)		
Race and ethnicity^c^				
African American	0	8 (6.0)	.96	
White	38 (92.6)	114 (85.7)		
Other or unknown	3 (7.3)	11 (8.3)		
Sexually active				
Yes	34 (82.9)	109 (81.9)	.66	
No	6 (14.6)	24 (18.1)		
Most bothersome symptom^d^				
Pain with vaginal penetration	23 (56.1)	74 (42.1)	.73	
Vaginal dryness	7 (17.1)	31 (23.3)		
Vulvar or vaginal itching	5 (12.2)	10 (7.5)		
Vulvar or vaginal irritation	4 (9.8)	9 (6.8)		
Vulvar or vaginal soreness	1 (2.4)	6 (4.5)		
Vaginal pH				
≤5	1 (2.4)	21 (15.8)	.05	
>5	40 (97.6)	111 (83.5)		
Vaginal Maturation Index, % superficial cells				
≤5	34 (82.9)	109 (82.0)	.29	
>5	1 (2.4)	10 (7.5)		
MENQOL total score^e^				
<Median (<3.16)	28 (68.3)	55 (41.4)	.002	
≥Median	11 (26.8)	73 (54.9)		
PHQ-9 depression score^f^				
No symptoms (<5)	33 (80.5)	88 (66.2)	.05	
Mild or worse symptoms	7 (17.1)	45 (33.8)		
GAD-7 anxiety score^g^				
No symptoms (<5)	25 (60.9)	83 (62.4)	.99	
Mild or worse symptoms	15 (36.5)	50 (37.6)		
ISI score^h^				
No symptoms (<8)	24 (58.5)	68 (51.1)	.33	
Mild or worse symptoms	16 (39.0)	65 (48.9)		

Abbreviations: BMI, body mass index (calculated as weight in kilograms divided by height in meters squared); GAD-7, 7-item Generalized Anxiety Disorder; ISI, Insomnia Severity Index; MENQOL, Menopause Quality of Life; PHQ-9, 9-item Patient Health Questionnaire; SHBG, sex hormone–binding globulin.

SI conversion: to convert estradiol to picomoles per liter, multiply by 3.671; estrone to picomoles per liter, multiply by 3.698; SHBG to nanomoles per liter, multiply by 8.896.

^a^ Sums may differ and percentages may not add up to 100% due to missing characteristic data.

^b^ *P* value from a logistic regression model of week 12 estradiol higher than 2.7 pg/mL vs lower than or equal to 2.7 pg/mL as a function of the participant characteristic of interest.

^c^ Other includes Hispanic, American Indian or Alaska Native, Asian or Pacific Islander, or unknown. Groups were combined due to small numbers. *P* value compares race as White vs African American, Hispanic, American Indian or Alaska Native, Asian or Pacific Islander, or unknown.

^d^ *P* value compares pain with vaginal penetration vs all other symptoms.

^e^ A higher MENQOL score is considered worse quality of life (range, 1-8).^15^

^f^ The PHQ-9 measures depression. A PHQ-9 score less than 5 indicates no depression (range, 0-20); we eliminated 1 item (suicidality).^16^

^g^ The GAD-7 scale measures anxiety. A GAD-7 score less than 5 indicates no anxiety (range, 0-17).^18^

^h^ The ISI measures insomnia. An ISI score less than 8 indicates no insomnia (range, 0-27).^19^

We then evaluated whether baseline estradiol levels, BMI, or years since menopause modified the response to treatment assignment. Associations between treatment group and week 12 estradiol concentrations did not significantly vary since menopause across participant subsets according to baseline estradiol levels, BMI, and years since menopause (Table 4).

**Table 4.** Associations Between Treatment Group and Week 12 Estradiol Concentrations by Baseline Factor Subgroups

Subgroup	No.	Vaginal estrogen vs placebo, % difference (95% CI)^a^	*P* value for interaction^b^
All participants	174	23.8 (6.9 to 43.3)	
Baseline estradiol, pg/mL			
<3	59	16.3 (−6.9 to 45.4)	.32
3 to <4	52	36.2 (7.2 to 73.0)	
≥4	63	2.3 (−17.4 to 26.7)	
BMI			
<25	82	27.6 (2.9 to 58.1)	.78
25 to <30	54	20.3 (−8.2 to 57.6)	
≥30	38	23.2 (−10.0 to 68.5)	
Time since menopause, y			
<5	25	11.7 (−27.6 to 72.2)	.14
5 to <10	54	10.6 (−15.2 to 44.1)	
≥10	93	40.3 (13.9 to 72.7)	

Abbreviation: BMI, body mass index (calculated as weight in kilograms divided by height in meters squared).

SI conversion: to convert estradiol to picomoles per liter, multiply by 3.671.

^a^ Percent difference derived by exponentiating estimates.

^b^ Estimates and 95% CIs for treatment group effect were calculated from linear models of log-transformed week 12 estradiol concentration as a function of treatment group, the subgroup of interest, and their interaction; *P* values for interaction were calculated from a separate linear model with log-transformed week 12 estradiol concentration as a function of treatment group, linear trend over the subgroup of interest, and their interaction. Models were adjusted for baseline estradiol level, clinical center, age, and BMI, as appropriate. Time since menopause subgroup models was not adjusted for age due to the close relationship between time since menopause and age.

## Discussion

Our analysis noted a clinically small, although statistically significant, increase in serum estradiol levels with 12 weeks of vaginal low-dose (10 µg) estradiol treatment. We also observed the probability of higher BMI as a contributor to higher circulating estrogen concentrations among women who are postmenopausal with moderate to severe vulvovaginal symptoms. Adjusting for age, BMI, and serum estradiol levels at enrollment did not attenuate the association between the use of vaginal estradiol, 10 µg, and increased serum estradiol levels. Using a cutoff value of 2.7 pg/mL, which was previously associated with a higher risk for breast cancer,^13^ we found no significant difference between the estrogen vs placebo groups in the proportion of women whose risk level changed from lower (≤2.7 pg/mL) to higher (>2.7 pg/mL).

Our trial studied a vaginal estradiol, 10-µg, tablet, which has been associated with lower serum estradiol concentrations than vaginal estradiol creams.^3,4^ One study of a 10-µg vaginal tablet showed an initial spike above 20 pg/mL in serum estradiol concentrations 8 hours after administration on the first day of use, but otherwise low concentrations through 83 days of use.^26^ A separate study noted increasing estradiol concentrations in the hours after administration with long-term use of a vaginal 10-µg tablet, but no serum estradiol concentrations higher than 10 pg/mL.^4^ A more recent study of a vaginal estradiol softgel also showed an initial concentration spike in the hours after first administration of the medication, but a lower spike after several weeks of treatment, and no significant increase in mean circulating estradiol concentrations.^2,27^ A more recent study suggested that there may be greater absorption of topical hormonal preparations initially, when the mucosa is thinner and more friable.^28^ We found a suggestion of greater influence of the vaginal estradiol use on serum estradiol concentrations in women with lower starting estradiol concentrations and longer duration since menopause, which would support the hypothesis that there is greater absorption with a thinner vaginal mucosa; however, the elevations in our trial were measured at 12 weeks. We could not assess initial transient spikes as have others.

We did not see changes in serum estrone or SHBG concentrations associated with vaginal estrogen vs placebo treatment assignment, which suggests a very limited systemic biological effect. After menopause, concentrations of estrone were higher than those of estradiol, as seen in our study, and have also been associated with to risk for breast cancer.^29^ Estrone concentrations are an indicator of estradiol concentrations—an association we also noted.^30^ In 1 study comparing the use of oral estradiol and a vaginal conjugated estrogen cream, a larger increase in serum estrone concentration was seen than in serum estradiol concentration for both formulations.^31^ Women using oral estradiol hormone therapy generally have an increase in SHBG levels, but we did not see any association between vaginal treatment and concentrations of SHBG.^16^ The lack of change in circulating estrone and SHBG concentrations suggests that the statistically significant increase in serum concentrations of estradiol seen in our study likely had little physiologic effect.

There are few data to guide identification of a safe concentration of serum estradiol in women who are postmenopausal. A single study of raloxifene demonstrated a lower risk of breast cancer only in women with serum concentrations of estradiol higher than 2.7 pg/mL and, in the placebo group, found a significantly greater incidence of breast cancer among individuals with concentrations above that threshold, suggesting that lower concentrations of estradiol may not contribute to breast cancer risk.^32^ However, that study used immunoassays to measure estradiol, which are likely to be less accurate at the lower concentrations seen in women who are postmenopausal. Our study used a highly sensitive LC-MS/MS assay certified by the CDC HoSt Program that had a high level of precision and accuracy in the low range. It is possible that concentrations are not comparable between the 2 studies. Meta-analyses, mostly including studies that used immunoassays, suggest that higher concentrations of serum estradiol in women who are postmenopausal are associated with greater risks for multiple adverse health outcomes, including breast cancer, thromboembolic disease, and heart disease. Multiple cohort analyses have found no significant associations between use of low-dose vaginal estradiol and any of these outcomes, suggesting that the small increases in serum estradiol concentrations seen in our cohort are not clinically significant.^6,7,12^ However, to our knowledge, no randomized clinical trials have specifically tested this claim.

### Limitations

This study has limitations. One of the primary limitations of our study and similar studies is the lack of a clear cutoff threshold for what concentration of circulating estradiol defines an increased risk for adverse outcomes. Our analysis used the cutoff of 2.7 pg/mL, which is based on a single study from 2002.^13^ Assays have changed dramatically since the publication of that study, and interpretation of all findings should take into account the newer ultrasensitive assays used in our studies. For these reasons, it is unlikely that this cutoff point is an appropriate marker for clinical risk with modern assays. Our study participants were part of a randomized clinical trial, which mitigates the impact of unmeasured confounders, but we did not have sufficient prevalence to assess potential confounders, such as alcohol use or smoking.

## Conclusions

Many patients and health care professionals have concerns about the use of estrogen-containing therapies after menopause and do not distinguish between low-dose vaginal formulations and systemic hormone therapy. Definitively answering the question of whether low-dose vaginal estrogen preparations are safe would require a very large randomized clinical trial. However, our analysis, in combination with findings from multiple previous cohort studies,^6,7,8,9,10^ suggests that vaginal use of estrogen preparations does not substantially alter serum concentrations, and adds to the evidence that any changes in serum estrogen concentrations likely have limited clinical significance.

## Notes

Supplement 1.
Trial Protocol

Supplement 2. **eTable 1.** Associations Between Baseline Participant Characteristics and Week 12 Estrone Concentration **eTable 2.** Associations Between Baseline Participant Characteristics and Week 12 SHBG Concentration **eFigure 1.** CONSORT Diagram of How Original Trial Participants Were Enrolled and How This Subset Was Selected **eFigure 2.** Serum Concentrations of Estrone at Enrollment and 12 Weeks for Participants in the MsFLASH Vaginal Health Randomized Trial Assigned to the Vaginal 10 μg Estradiol Tablet + Placebo Gel (n = 88) or Dual Placebo (n = 86) Arms **eFigure 3.** Serum Concentrations of Sex Hormone-Binding Globulin (SHBG) at Enrollment and 12 Weeks for Participants in the MsFLASH Vaginal Health Randomized Trial Assigned to the Vaginal 10 μg Estradiol Tablet + Placebo Gel (n = 88) or Dual Placebo (n = 86) Arms

Supplement 3.
Data Sharing Statement
