# Supplementary material for: Association of Vaginal Estradiol Tablet With Serum Estrogen Levels in Women Who Are Postmenopausal: Secondary Analysis of a Randomized Clinical Trial
Source: JAMA Netw Open. 2022 Nov 14;5(11):e2241743. doi: 10.1001/jamanetworkopen.2022.41743 (PMC9664265; doi:10.1001/jamanetworkopen.2022.41743)
Supplement: Supplement 1. — Trial Protocol [file jamanetwopen-e2241743-s001.pdf]

MsFLASH: The Vaginal Health Trial
Protocol Version 1.6

Section	Topic	Page
1.	Overview and Schema	3
2.	Objectives	3
3.	Background	4 - 5
4.	Study Population	6
4.1	Inclusion Criteria	6
4.2	Exclusion Criteria	7
5.	Intervention	7
5.1	Vagifem®	7
5.2	Replens®	7
5.3	Placebo	7
6.	Randomization and Blinding	7
7.	Outcomes	8
8.	Study Plan	9
8.1	Recruitment	9
8.2	Informed Consent	9
8.3	Screening, Randomization, and Study Visits	10 - 13
8.4	Early Termination	13
8.5	Adherence and Retention	14
8.6	Participation Payments	14
9.	Data Collection	14
9.1	Primary Outcome	15
9.2	Secondary Outcomes	15
9.3	MsFLASH Common Measures and Other Measures of Interest	16
9.4	Pilot Translational Studies	17 - 19
10.	Statistical Considerations	19
10.1	Sample Size and Power	19
10.2	Statistical Analysis	20
11.	Study Operations	20
11.1	Data Management	20
11.2	Specimen Handling	21
11.3	Packaging of Study Medications and Placebo	22
11.4	Quality Control	22 - 23
11.5	Quality Assurance Site Visits	23
12.	Study Monitoring	24
12.1	General	24
12.2	Accrual, Adherence, Retention	24
12.3	Safety Monitoring Plan (Adverse Events and Serious Adverse Events)	24
12.4	Vagifem® Safety and Potential Risks	25
12.5	Replens Safety and Potential Risks	25
12.6	Placebo Vaginal Tablet and Gel Safety and Potential Risks	25
12.7	Adverse Event Monitoring and Management Related to Study Drugs	26
12.8	Vaginal Specimen Collection Safety and Potential Risks	26
12.9	Venipuncture Safety and Potential Risks	27
12.10	Other Safety Issues	27
12.11	Trial Monitoring (DSMB)	27
13.	Study Timeline	27
14.	Summary of MsFLASH Network	27
15.	References	28 - 34

MsFLASH Vaginal Health Trial Organization
Guthrie (Fred Hutchinson Cancer Research Center), LaCroix (University of California San Diego), Reed (University of Washington) – Multi-PIs
Protocol PI – Reed (University of Washington), Mitchell (Massachusetts General Hospital)<br>Clinic Site PIs: Reed/Newton (Group Health Research Institute), Ensrud/Diem (University of Minnesota)<br>Biorepository and Laboratory: Fredricks (Fred Hutchinson Cancer Research Center), Mitchell (Massachusetts General Hospital)

Investigators:

Katherine A. Guthrie, PhD, MsFLASH Data Coordinating Center, Fred Hutchinson Cancer Research Center (FHCRC), Seattle, WA

Andrea Z. LaCroix, PhD, University of California San Diego (UCSD), San Diego, CA

Susan D. Reed, MD, University of Washington (UW), Seattle, WA

Caroline Mitchell, MD, Massachusetts General Hospital (MGH) and Harvard Medical School (HU), Boston, MA

Katherine Newton, PhD, Group Health Research Institute (GHRI), Seattle, WA

Kristine Ensrud, MD, Susan Diem, MD, University of Minnesota, Minneapolis, MN

David Fredricks, MD, Fredricks Lab, Fred Hutchinson Cancer Research Center, Seattle, WA

Consultants:

Nancy F. Woods, University of Washington, Seattle, WA

Stacey T. Lindau, University of Chicago, Chicago, IL

Jan L. Shifren, Massachusetts General Hospital and Harvard Medical School, Boston, MA

Julia R. Heiman, Indiana University, Bloomington, IN

Janet Carpenter, Indiana University, Bloomington, IN

In addition to the above institutions, Sharp Clinical Services will package and blind study drug.

Study Sponsor: National Institutes of Health/National Institute of Aging (NIA)

1. Overview and Schema

The MsFLASH05 *Vaginal Health Trial,* is a randomized, double-blind, placebo-controlled, three-arm clinical trial of postmenopausal women aged 45-70. The design includes 12 weeks of treatment with an estradiol vaginal tablet plus placebo gel, hydrophilic moisturizing vaginal gel plus placebo vaginal tablet, or double placebo (gel and tablet). Participants attend three clinic visits: one to conduct randomization and collect baseline measures, and two to collect follow-up data at 4 and 12 weeks.

This study is part of a new application from the MsFLASH *(Menopause Strategies: Finding Lasting Answers for Symptoms and Health)* network, a group of investigators performing clinical trials designed to find new ways to alleviate the most common, bothersome symptoms of the menopausal transition and postmenopause. Four randomized controlled clinical trials, and several ancillary and pilot projects, were completed during the initial funding period of the MsFLASH network.

This protocol describes the first planned randomized controlled trial of the second funding period; it is designed to test the efficacy of both a vaginal estradiol tablet and a vaginal gel in alleviating vaginal symptoms. See Figure 1, Overall Study Design.

Figure 1.

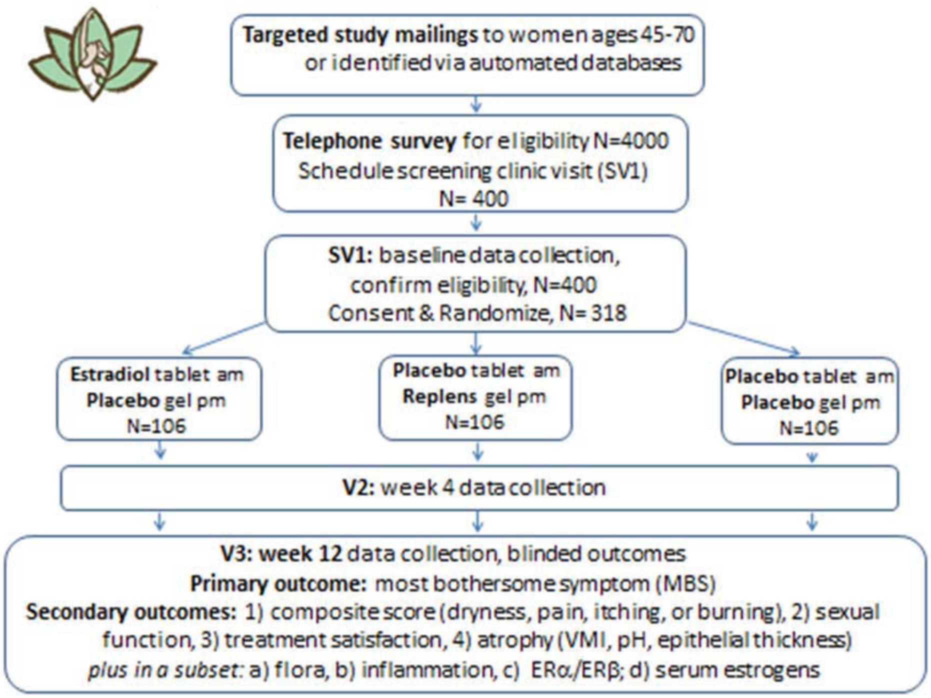

2. Objectives

The primary objective of this trial is to determine the efficacy of an ultra-low dose hormonal vaginal tablet (Vagifem®) and a non-hormonal vaginal gel (Replens®) in relieving vaginal symptoms.

The specific study aims are:

Primary Specific Aim 1*:* to compare the effects of: 1) Vagifem® plus placebo gel vs. placebo tablet plus placebo gel, and 2) Replens® plus placebo tablet vs. placebo gel and placebo tablet *on the primary outcome* – change from baseline to 12 weeks in severity of the most bothersome symptom (MBS): dryness, itching, irritation, soreness and dyspareunia.

Primary Hypotheses Aim 1: Vagifem® 10 mcg is superior to placebo, and Replens® is superior to placebo in improving severity of the most bothersome symptom

Secondary Specific Aim 1.1: Evaluate the relative effectiveness of Vagifem® vs. Replens® in reducing the severity of the most bothersome symptom.

Secondary Hypotheses Aim 1.1: Vagifem® 10 mcg is superior to Replens® in improving severity of the most bothersome symptom.

Secondary Specific Aim 1.2: Evaluate the impact of active treatment (Vagifem® or Replens®) relative to placebo *on secondary outcomes* – change from baseline to 12 weeks in:

a) composite vaginal symptom index (VSI) score including dryness, itching, irritation, soreness among all women, and, dyspareunia among sexually active women;
b) sexual function;
c) treatment satisfaction;
d) menopause quality of life; and
e) objective measures of GU atrophy (pH, vaginal maturation index (both sites) and epithelial thickness (Seattle site only).

Secondary Hypotheses Aim 1.2: Vagifem® 10 mcg is superior to placebo, and Replens® is superior to placebo in improving composite vaginal symptom index over 12 weeks, sexual function, overall satisfaction, menopausal quality of life, vaginal pH, thickness and vaginal maturation index (VMI) over 12 weeks of treatment.

Secondary Specific Aim 1.3: Develop a biorepository of specimens to allow translational, mechanistic, systems biology studies of the etiology of vaginal symptoms.

Secondary Specific Aim 1.4: Perform additional novel exploratory analyses in a subset of women (n=75) to *evaluate secondary outcomes*:

a) the postmenopausal vaginal microbiome;
b) vaginal mucosal inflammation;
c) hormones (serum concentrations and vaginal receptor expression) as underlying mechanisms of treatment response; and
d) the postmenopausal genitourinary and rectal microbiomes.

Secondary Hypotheses Aim 1.4: Menopausal vaginitis symptoms are not simply due to low serum estrogen levels, but rather to alterations in the vaginal microbiota and/or the mucosal immune response (Figure 2).

Figure 2. Hypothesized pathophysiology of menopausal vaginitis

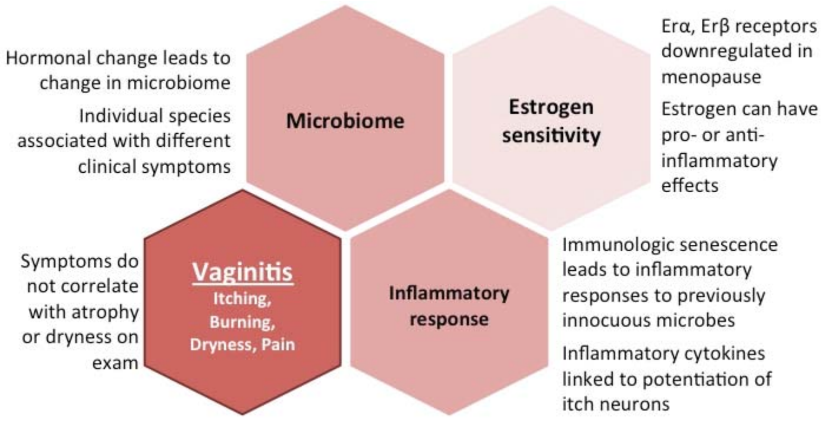

We will use the framework of the proposed clinical trial to collect samples and to refine the design of translational studies to test this set of hypotheses. The biorepository will include: blood, rectal swabs, vaginal swabs, vaginal cytobrush, cervicovaginal lavage, and vaginal biopsy tissue. The biorepository will be maintained at the Fred Hutchinson Cancer Research Center, which has 24-hour monitoring of freezer integrity. Several pilot studies are proposed to facilitate adequate design and execution of future translational studies (described in Section 9.4).

3. Background

Bothersome postmenopausal vaginal symptoms are prevalent and adversely affect quality of life, including sexual function. Up to 40% of postmenopausal women have vaginal symptoms presumed to be related to vaginal atrophy.[1] Similar to premenopausal women, postmenopausal women with vaginal inflammation or vaginitis may present with discharge, itching, irritation, and pain or dyspareunia (38%) but also commonly report vaginal dryness (75%).[1] Despite this high prevalence of postmenopausal vaginal symptoms, data to guide treatment choices is extremely limited. Minimal data supports efficacy of nonprescription treatments for vaginal dryness and symptoms, but their comparative efficacy versus estrogen therapy is unknown. There is a paucity of data concerning the microbiologic, inflammatory and hormonal mechanisms underlying postmenopausal vaginal symptoms and their impact in determining treatment response in uncertain.

Vaginal health research has concentrated on premenopausal women in the past. Other than the role of diminished estrogen on genitourinary atrophy, very little is understood about the causes or treatment of postmenopausal vaginal symptoms with advancing age. The number of US women over age 65 is expected to

double by 2022 and approximately 40% will experience bothersome vaginal symptoms,[1-5] 75% may have vaginal dryness and up to 40% may experience pain with intercourse. With new therapies for male sexual dysfunction, female partners with genitourinary (GU) atrophy are more apt to need treatment for vaginal symptoms to maintain partner intimacy without pain. Despite a high prevalence of postmenopausal vaginal symptoms, data to guide treatment choices is extremely limited.

Treating vaginal symptoms, including dyspareunia, is the first step in addressing sexual dysfunction. Sexual function in aging women is woefully understudied, although evidence suggests that postmenopausal vaginal symptoms (in particular dyspareunia) adversely affect sexual function.[1,6,7] Genitourinary (GU) atrophy is related to postmenopausal vaginal symptoms and diminished female sexual function in some, but not all women. GU atrophy, diagnosed by findings on physical examination and VMI, usually manifests several years after the final menstrual period. GU atrophy is directly mediated by hypo-estrogenism resulting in loss of collagen, and thinning and drying of the vaginal epithelium.[8] The chronicity of suppressed estrogen concentrations in postmenopausal woman may add to diminished nerve conduction, diminished vasoconstriction and vasodilation control[6] that ultimately contribute to the rise in vaginal symptoms in the postmenopausal years. However, while the overwhelming majority of postmenopausal women have GU atrophy 5 years or more from their final menstrual period, not all women with GU atrophy have bothersome symptoms.[9,10]

The Estradiol hemihydrate vaginal tablet (Vagifem® 10 mcg) is an affordable, popular treatment for vaginal symptoms. In women with postmenopausal vaginal dryness, itching, pain or burning, meta-analyses of past randomized trials conclude that local estrogen creams reduce symptoms (particularly dryness) in the majority of women.[11,12] While creams, often with substantial systemic absorption, remain the mainstay of current therapies, only 30% of postmenopausal women prescribed vaginal creams use them beyond 6 months.[13] Improved treatment compliance, satisfaction, and adherence are observed with estrogen tablets or rings over creams, though insertion of rings is problematic for some women.[13,14] Thus, vaginal tablets may be a preferable treatment option for many women.

Non-hormonal hydrophilic vaginal gel (Replens®) is a popular over-the-counter product for vaginal dryness. This and similar non-hormonal products are touted as attractive options for women with bothersome postmenopausal vaginal symptoms and are recommended by the American College of Obstetrics and Gynecology,[15] NAMS,[1] the American Academy of Family Physicians,[16] and the International Menopause Society.[6] However, little data exists to support the view that postmenopausal vaginal symptoms improve with use of non-hormonal therapies, and some *in-vitro* studies suggest one of the most commonly used products, Replens®, a polycarbophil hydrophilic moisturizer, may even be harmful to mucosal integrity.[17] To our knowledge, no blinded randomized placebo-controlled trial has evaluated the efficacy of vaginal gels on postmenopausal vaginal symptoms. Head to head, adequately powered, double-blind, placebo controlled trials comparing ultra-low-dose local estrogen therapy to a commonly used non-hormonal therapy are needed to inform women about therapeutic efficacy.

We selected the severity of the most bothersome symptom (MBS) as our *primary outcome* because it is one of the FDA-recommended outcomes for trials of treatment for menopausal symptoms.[18] Additionally, preliminary data from MsFLASH 03 showed that most women complain of a single severe symptom (most commonly dryness), and we are concerned that a composite score may dilute the outcome. We chose a composite Vaginal Symptom Index (VSI, scale 0-3, average of 4 items: dryness, itching, irritation, soreness; scale 0=none, 3=severe) as a secondary outcome [19] to reflect the breadth and complexity of women's symptoms. Trials conducted by the pharmaceutical industry use the FDA recommended outcomes,[18] and also measures of GU atrophy pH and VMI.[20-24] We include measures of GU atrophy such as pH and VMI, [20-24] which have been used in prior trials, as secondary outcomes.

Vaginal health is intricately bound to the vaginal microbiome.[25-27] Yet little is known about healthy vaginal aging, normal postmenopausal microflora, or the interaction of GU atrophy with a disturbed microflora and an aging immune system.[28-30] Microbial communities colonize mucosal surfaces in the gut, the mouth, and the vagina[31] and play a major role in our susceptibility to disease. For example, gastrointestinal bacteria interact with the gut epithelium to create a balance of immune tolerance and primed immune response. Failure of this system has been implicated in the development of inflammatory bowel diseases. In the premenopausal vagina, Lactobacillus species predominate among a multitude of bacterial species, and maintain an acidic environment associated with normal reproductive health.[32-34] Alteration in this microbial community has been associated with inflammation[35] and vaginal irritation.[27] After menopause and with aging, the composition of the vaginal microbiome changes,[36-38] but little is known about how perturbations in vaginal flora, including *Lactobacillus*

species, immune response, or the presence of specific vaginal epithelial hormone receptors (ERα or ERβ) impact risks for postmenopausal vaginal symptoms. Additionally, the composition of the gastrointestinal microbiota may influence circulating hormone levels[39] or the composition of the vaginal microbiota.[40]

4. Study Population

The target population will include women in general good health, aged 45–70 years, who report bothersome vaginal symptoms. The study will enroll 318 women across two sites (Seattle and Minneapolis), and utilize a third site for recruitment if needed (Boston).

4.1 Inclusion Criteria

- Females aged 45-70 years
- 2 or more years since last natural menstrual period, or surgical menopause (bilateral oophorectomy)
- At least 1 vaginal symptom (inside or outside the vagina) reported from the following list, experienced in the past 30 days which is moderate or severe:
  - Dryness at least once a week
  - Itching at least once a week
  - Irritation at least once a week
  - Soreness/pain at least once a week
  - Pain associated with sexual activity at least once
- Signed informed consent

4.2 Exclusion Criteria

- Current unexplained abnormal genital bleeding (or any unevaluated bleeding since menopause)
- Currently pregnant, attempting pregnancy or breast feeding.
- Current acute vaginal infection (as indicated by wet mount at V1)
- Pelvic or vaginal surgery in prior 60 days
- Antibiotic use in the past 30 days
- Women under age 55 with endometrial ablation
- Women under age 55 with hysterectomy and at least one ovary
- Current cancer treatment (exception basal or squamous skin cell cancers)
- Current or past thromboembolic disease (pulmonary embolus or deep vein thrombosis, not including thrombophlebitis), myocardial infarction, or stroke
- Current severe liver disease
- Current or past breast or endometrial cancer or pre-cancer
- Blood clotting disorder (e.g., Factor V Leiden, prothrombin mutation, protein C, protein S or antithrombin deficiency)
- Porphyria
- Current or past lichen sclerosus or lichen planus
- History of adverse reaction to vaginal estrogen or Replens
- Use of any systemic reproductive hormones (hormonal contraception, postmenopausal hormone therapies, SERMS) in past 2 months
- Use of hormonal contraception in the past year
- Use of any type of vaginal estrogen product, (however, interested women will be allowed to join the study if they abstain from use during the month preceding enrollment)
- Use of any vaginal moisturizer, stimulatory lubricant, douche, vaginal prebiotic or probiotic, or soap in the vagina in past 1 month (however, interested women will be allowed to join the study if they abstain from use during the month preceding enrollment)
- Unwilling to abstain from use of any non-study vaginal estrogen, vaginal moisturizer, stimulatory lubricant, douche, vaginal prebiotic or probiotic, or soap in the vagina throughout the trial
- Unable to follow instructions, complete questionnaires, or physically unable to place product in the vagina
- Current participation in another drug trial or intervention study
- Chronic vulvovaginal symptoms in the 5 years before menopause (defined as a vaginal or vulvar condition requiring more than 4 visits to a health care provider in a given year)

5. Intervention

Participants (n=318) will be randomly assigned to one of three 12-week, double-blinded treatments:

1) Estradiol 10 mcg vaginal tablet daily (Vagifem®) (am) for 2 weeks, then 2 days a week (pm) for 10 weeks and placebo vaginal gel every 3 days (pm) for 12 weeks
2) Placebo vaginal tablet daily (am) for 2 weeks, then 2 days/week (pm) for 10 weeks and hydrophilic moisturizing vaginal gel (Replens®) every 3 days (pm) for 12 weeks
3) Placebo vaginal tablet daily (am) for 2 weeks, then 2 days/week (pm) for 10 weeks and placebo vaginal gel every 3 days (pm) for 12 weeks

The appearance, packaging, and bottling for the active and placebo tablets and gel will be identical to maintain the double-blind throughout the 12-week trial. At the time that study data are released, participants will be informed of their study treatment assignment by mail.

5.1 Vagifem®

One hundred six participants will be randomized into the Vagifem® arm of the study. Women in this arm will be given a estradiol 10 mcg vaginal tablet (Vagifem®) daily (am) for 2 weeks, then 2 days/week (pm) for 10 weeks and placebo vaginal gel (hydroxyethylcellulose gel) every 3 days (pm) for 12 weeks. Each woman will be provided with 2 reusable vaginal tablet applicators and 2 reusable gel applicators.

Vagifem® 10 mcg tablets are small white film-coated tablets.[6] Each tablet is placed using an applicator. The active ingredient for a 10 mcg tablet is 10.3 mcg of estradiol hemihydrate equivalent to 10 mcg of estradiol. The coating is made of hypromellose and polyethylene glycol. The excipient (inactive) ingredients are hypromellose, lactose monohydrate, maize starch, and magnesium stearate.

5.2 Replens®

One hundred six participants will be randomized into the Replens® arm of the study. This arm is comprised of hydrophilic moisturizing vaginal gel (Replens®) every 3 days (pm) for 12 weeks and placebo vaginal tablet daily (am) for 2 weeks, then 2 days/week (pm) for 10 weeks. Each woman will be provided with 2 reusable vaginal tablet applicators and 2 reusable gel applicators.

Replens® is a bioadhesive polycarbophil-based vaginal gel.[41,42] The ingredients are purified water (vehicle humectant), glycerin (moisturizer), mineral oil (Paraffinum Liquidum as a moisturizer), polycarbophil and carbomer homopolymer type B (allow the product to stick to the vaginal wall), hydrogenated palm oil glyceride (moisturizer), sorbic acid (preservative, antimicrobial), methylparaben (only in the 35 gm tubes), sodium hydroxide (adjusts pH of product so it is suitable for vaginal use). Replens® does not contain any animal derivatives. It is safe for daily use.

5.3 Placebo

One hundred six participants will be randomized into the placebo arm of the study. This arm is comprised of two placebo preparations: a placebo vaginal tablet and placebo vaginal gel. Women will be given a placebo vaginal tablet daily (am) for 2 weeks, then 2 days/week (pm) for 10 weeks and a placebo vaginal gel (hydroxyethylcellulose gel) every 3 days (pm) for 12 weeks. Each woman will be provided 2 reusable vaginal tablet applicators and 2 reusable gel applicators.

Placebo vaginal tablet. The placebo tablet coating and excipient ingredients will be the same as are used for Vagifem®. The coating will be hydroxypropyl methylcellulose (hypromellose) and polyethylene glycol. The excipients will be hypromellose, lactose monohydrate, maize starch, and magnesium stearate.

Placebo vaginal gel. The product is a hydroxyethylcellulose gel.[43,44]

6. Randomization and Blinding

Participants who meet all study eligibility criteria will be randomized in a 1:1:1 ratio to estradiol vaginal tablet (Vagifem®), hydrophilic moisturizing vaginal gel (Replens®), or placebo. Prior to randomization, all key eligibility data will be entered into the centralized MsFLASH database. Clinic staff will then execute a database function confirming eligibility. Once confirmed, the randomization assignment will be made, based on a dynamic allocation scheme, stratified by site. The database will identify the appropriate drug box from a numbered carton. Clinic staff will retrieve the box, attach a participant label to all products in the box, enter the box ID into the database to verify it was dispensed to the participant and record the box number in the

participant's chart. Study participants and all clinical staff who have contact with participants will be blinded to the randomization assignment of all participants.

7. Outcomes

The primary and secondary outcome measurements for the Vaginal Health Trial are listed in Table 1. All outcomes will be measured at baseline, week 4, and week 12, including:

<u>Primary Outcome</u>: Mean change from baseline to 12 weeks in the severity of most bothersome symptom (MBS) on a scale of 0-3, better to worse.

<u>Secondary Outcomes</u>: 1) Satisfaction with treatment (Likert scale and Patient Benefit Evaluation) at follow-up; and mean change from base-line to 12 weeks in: 2) composite Vaginal Symptoms Index (VSI). [The VSI is a Modified Bachman scale[19] measuring vaginal dryness, itching, irritation, soreness, and pain with sexual activity (among sexually active women), each rated 0=none to 3=severe, and then averaged for a total score of 0-3] 3) Female Sexual Function Index (FSFI) pain domain (scale 0-6, worse to better) among sexually active women and FSFI composite (scale 2-36) among all women, Sexual Function Distress Item 1 (FSDS-R) (scale 0-4, better to worse); 4) Menopausal quality of life (MENQOL) (scale 1-8, better to worse); 5) pH (<5; >5); 6) VMI (% parabasal, intermediate, and superficial cells) and 7) epithelial thickness (Seattle site only).

Table 1: Study-Specific Measures – Primary and Secondary Outcomes

Measure	Description	Details on Measurement		Weeks
**Vaginal Symptoms**	*VSI Composite*[19,26,45]*:* dryness, itching, irritation, or soreness, and dyspareunia in sexually active women	Self-report	Scale 0-3, average of 4 items, maximum score 3	Weekly + Daily weeks 0, 4,12
	MBS Severity of most bothersome symptom: Vaginal dryness, itching, irritation, soreness or dyspareunia[18,23]	Self-report	Most bothersome symptom (MBS), range 0-3 (0=none, 1=mild, 2=moderate, 3 = severe)	Weekly + Daily weeks 0, 4,12
**Treatment satisfaction**	Likert scale	Self-report	Scale 0= no to10= complete satisfaction	Weeks 12
	Patient Benefit Evaluation (PBE)	Self-report	Overall, do you believe that you experienced a meaningful benefit from the study medication? (yes/no)	Week 12
**FSFI**[46-48]<br><br>**FSDS-R**[48]	Assess sexual function, dyspareunia, sexual function distress	Self-report	Composite score= 2 (not sexually active and no desire) to 36, 6 domains<br>Distress –Item 1, scale 0-4, If answer >0, complete remaining 12 items	Weeks 0, 4,12
**MENQOL**[49,50]	Assess menopause QOL	Self-report	Range 1-8, 29 items	Weeks 0, 4,12
**Pelvic examination**	Assess for infection and to grade GUA	Physical examination	Assess color, rugosity, moisture, integrity, pain	Weeks 0, 4,12
**Yeast culture**	Assess for yeast	Vaginal swab	Presence of yeast	Weeks 0, 4
**Nugent Score**[51]	Assess for vaginosis	Vaginal swab, slide	Presence of lactobacillus, gram variable rods, score 0-10	Weeks 0, 4, 12
**Vaginal pH**[18]	Acid base status of vagina	Vaginal swab	Evaluate pH < 5.0, $\geq$ 5.0	Weeks 0, 4,12
**Vaginal Maturation Index**[18]	Evaluate epithelial atrophy	Vaginal spatula	Maturation index to assess % of cells: superficial, intermediate and parabasal	Weeks 0,12
**Vaginal biopsy (Seattle site only)**	Assess epithelial thickness	Biopsy	Epithelial thickness will be measured	Weeks 0, 4,12
	Evaluate estrogen receptors (ER), inflammation	Biopsy	Immunohistochemistry ER	Weeks 0,12
**Vaginal Microbiome**[52-56]	Microbiology of the vagina	Vaginal swab	16S rRNA gene PCR with Pyrosequencing, Illumina high throughput sequencing, metabolomics	Weekly + Daily weeks 0, 4,12

**Serum hormones**	Serum estradiol	Serum	LC/SRM/MS	Weeks 0,12
**Vaginal Inflammatory Markers**[57-59]	Inflammatory markers	Lavage, cytobrush	mRNA, Vaginal lavage fluid proteins, IHC immune proteins	Weeks 0, 4,12

Other Measures of Interest: vaginal symptoms (Day-to-Day Impact of Vaginal Aging symptom questionnaire (DIVA),[60] insomnia symptoms, Insomnia Severity Index (ISI),[64] depression Patient Health Questionnaire (PHQ-8),[63] anxiety Generalized Anxiety Disorder (GAD-7),[62] urinary incontinence, Incontinence Severity Index (ISI),[65] and the 3-Incontinence Questions Tool (3IQ),[66] bladder pain, a history of recurrent urinary tract infections, rectal swab collection for biorepository and intake of prebiotics and probiotics.

In addition, a series of pilot translational studies will include analysis of biorepository specimens from 25 randomly selected women from each treatment arm (total 75 women). Vaginal and rectal microbiome, reproductive hormone, and immunology analyses are described in Section 9.4.

8. Study Plan

The recruitment, retention, sample sizes, successful completion, and cost-efficiencies of the MsFLASH research protocols rely on the networked operational design described below (Table 2).

8.1 Recruitment

Recruitment will occur primarily through targeted mailings to potential participants and screening will occur via phone calls. Targeted mailings will include an information letter or postcard with a recruitment phone number and website address. As previously implemented in other MsFLASH trials, Kaiser will execute all mailings and Group Health Research Institute will conduct phone screen activities. Repeat mailings will be sent up to two times, depending on initial response. Two sites (Seattle and Minneapolis) will recruit participants for this study, with a third site (Boston) available if needed to reach recruitment targets. All sites have access to large populations from which to recruit. Electronic health records (EHR) will be used to identify age-eligible women who are members of Group Health in Seattle. Purchased mailing lists will be used for recruitment in Minneapolis and as needed in Seattle. A pilot trial of recruitment via social media using the Facebook platform will be performed in the same population at both sites.

Study postcards to support recruitment efforts will be developed by the Data Coordinating Center **(**DCC), with input from the clinical Network sites. Sites may also develop site-specific materials. In preparation for the study, the Group Health clinical site will conduct two focus groups (about 20 women total) to discuss recruitment and recruitment materials. The purpose of the focus groups is to identify effective ways to invite women to join the vaginal health study, and thus design the best possible study invitation materials. Volunteers will be identified from Group Health electronic health records (HER), invited to participate by mail or phone call, take part in a 1.5-hour discussion, and provided with a snack, free parking, and $40 in compensation for their time. All recruitment materials, including mailings and advertisements, will be submitted to each participating site's Institutional Review Board (IRB) for review and approval prior to use at that institution.

8.2 Informed Consent

Consent to screen will be obtained verbally at the start of the screening phone call. Before the recruiter asks any of the screening questions, she will inform the potential participant that the purpose of the call is to begin the process of screening for eligibility and that all responses are confidential and completely voluntary. Continuation of the phone call and response to questions implies consent to the phone call screening. Women saying that they prefer not to answer questions will be thanked for their time and the phone call will end. Women may ask to stop the screening phone call at any time.

Written informed consent will be obtained at the first study visit prior to any other study procedures. Study staff will explain the purpose of the study, the procedures and treatments, the requirements of participation, risks and benefits, and the right to withdraw at any time, and answer any questions. The HIPAA consent will also be carefully reviewed with all participants.

All consent documents will be approved by the IRB of each participating network site and the DCC. Signed consent forms will be placed in the participant's administrative research file (as distinct from hardcopy research data files) and a copy will be given to the participant.

8.3 Screening, Randomization, and Study Visits

Eligibility screening occurs through a series of phone and in-person contacts. After eligible participants are randomized to a study arm, they will participate in two additional in-person visits (conducted after 4 and 12 weeks on treatment) and four phone contacts (conducted at 1, 3, 7, and 11 weeks post-randomization), as described in the section below. Table 2 summarizes the contacts and activities involved in screening, randomization, and study participation.

Table 2: Participant Study Activities

Stage	Location	Activity
Recruitment	Home	• Women mailed invitation letters and call study hotline if interested
Phone Eligibility Screen	Home	• GHRI Survey obtain verbal consent by phone and administer eligibility screen, including the Vaginal Symptom Index
Before Visit 1 (V1)	Home	• Eligible women mailed study consent and questionnaires<br>• Women complete Eligibility Questionnaire which includes a 3-day practice diary, and mail to the sites<br>• Sites review mailed questionnaires and confirm eligibility<br>• Sites contact eligible participants and schedule V1<br>• Participants instructed to complete Baseline Questionnaire and review consent prior to V1
Visit 1 (V1) Baseline	Research Clinic	• Staff reviews study consent Eligibility and Baseline Questionnaires with participant<br>• Instruct participant on single self-collection vaginal swab for wet mount<br>• Perform wet mount:<br>* If the wet mount is positive, the participant is ineligible at this visit. If positive for yeast, bacterial vaginosis (BV) or trichomoniasis, refer to provider for treatment. If positive for yeast, staff informs participant that over the counter treatment is an option. All women with positive wet mounts may return in 2 to 6 weeks for repeat wet mount (must be > 4 weeks for BV and trichomonas, to comply with antibiotic exclusions).<br>* If wet mount negative, and still eligible, enroll, perform pelvic exam, collect vaginal and rectal specimens and randomize.<br>• Vaginal biopsy if participant consents (Seattle site only)<br>• Non-fasting blood draw after normal wet mount<br>• Randomize, weigh, and dispense study medication after normal wet mount<br>• Measure height, weight
Weeks 0-12	Home	• Take medications as instructed<br>• Record daily bleeding, sexual activity, medications, lubricants, douche<br>• Complete Vaginal Symptom Diary and collect vaginal swabs daily weeks 0, 4,and 12 plus once weekly all other study weeks<br>• Mail in swabs and diaries weekly
Weeks 1, 3, 7, 11 and as needed	Phone	* Staff review with participants: medication instructions, answer questions, query about side effects, adherence, compliance, clinic visit reminders<br>* If participant fails 2 weeks of mailed specimens, an additional call is made
Week 4 Visit (V2)	Research Clinic	* Collect questionnaires from participants<br>* Pelvic examination, wet mount, vaginal and rectal specimens, biopsy if consents (Seattle site only)<br>* If wet mount positive for yeast, BV or trichomoniasis, refer to provider for treatment. If positive for yeast, staff informs participant that over the counter treatment is an option. All women remain in study on study drug<br>* Medication adherence: count Vagifem® tablets and weigh Replens® tube
Week 12 Visit (V3)	Research Clinic	* Collect questionnaires from participants<br>* Non-fasting blood draw<br>* Pelvic examination, wet mount, vaginal and rectal specimens, biopsy if consents (Seattle site only)<br>* If wet mount positive for yeast, BV or trichomoniasis, refer to provider for treatment.<br>* Medication adherence: count Vagifem® tablets and weigh Replens® tube

8.3.1 Eligibility Screen by Phone

The study recruitment calls for all sites will be conducted by the Group Health Survey department. Women who express interest in the study by responding to a recruitment mailing or flyer will be contacted by phone. The study will be briefly described, and women asked to provide verbal consent to screen for the study. Screening questions that will be asked include age, frequency and bother of vaginal symptoms, current use of hormones or vaginal products, menopausal status and medical conditions that are contraindications for the use of Vagifem.

Women who are eligible after the telephone screen and who verbally consent to further contact will be mailed a cover letter explaining more about the study eligibility process, eligibility and baseline questionnaires, and the consent. Women will be asked to complete the eligibility questionnaire, which includes a 3-day “practice diary”, and mail it to the sites.

The sites will review the mailed eligibility questionnaire and call eligible women to schedule a clinic visit. The women will be asked to review the baseline questionnaire, review the consent, and bring them to the first visit. Ineligible women will be called and thanked for their interest in the study. If further clarification of eligibility is needed, site study clinicians will contact potentially eligible women by telephone.

8.3.2 Baseline and Randomization Visit (V1)

At Baseline **Visit (V1)**, the following tasks occur:

- Informed consent for the study will be reviewed and signed.
- The Baseline questionnaire will be collected and reviewed for completeness. If the woman has not brought her completed questionnaire to the visit, she will be asked to complete one during the visit.
- Physical measurements, including height and weight will be obtained.
- A non-fasting blood sample for serum estrogen metabolite concentrations and biorepository will be collected.
- Participants will be instructed on how to self-collect a vaginal swab.
- Participants will self-collect a vaginal swab for wet mount, clinician will assess pH and wet mount for acute infection:
  - o If positive for yeast infection, bacterial vaginosis (BV), or trichomoniasis, refer to their provider for treatment. If positive for yeast, staff informs participant that over the counter treatment is an option. Women may return between 2-6 weeks for repeat wet mount. If treated for BV or trichomoniasis, must return > 4 weeks to comply with the antibiotic exclusion. If wet mount negative, continue with enrollment and randomization process. If positive, ineligible.
  - o If negative, the woman will proceed with pelvic examination, vaginal and rectal specimen collections.
- A vaginal fluid smear will also be made with the self-collected swab, for Nugent scoring.
- A standardized pelvic exam with documentation of vulvar and vaginal skin breaks, discoloration, amount of lubrication and discomfort and collection of vaginal samples for yeast culture, vaginal and rectal samples for the biorepository, and vaginal swab and cytobrush sampling to assess GU atrophy and inflammation will be conducted. A vaginal smear for Nugent score and spatula collection for Maturation Index will be made.
- A vaginal biopsy will be conducted for women who consented to the biopsy (Seattle site only).
- Randomization: Participants who consent and continue to meet all study eligibility criteria will be randomized to Vagifem®, Replens®, or placebo treatment.
- Study tablet and gel dispensation: Study pills will be dispensed in a double-blind fashion. Participants will be instructed to insert 1 study tablet vaginally in the morning daily for the first two weeks then twice a week in the evening for the next 10 weeks and the study gel vaginally every 3 days in the evening for 12 weeks. Ideally, the tablet and gel should be applied at least 12 hours apart.
- Participants will receive 2 containers, one with the tablets, and one with the gel, along with 2 types of reusable vaginal applicators (one for the tablet, one for the gel) and specific instructions, both verbal

and written, on how and when to use the study medications. For all medications (active or placebo), the tablet and gel containers will be identical and contain identical-looking medications. The database will identify the appropriate containers of study medications from a numbered carton that has been logged into the local inventory. This system will provide the clinic staff with the container numbers so that the appropriate containers will then be retrieved from a double-locked on-site storage space. The clinic staff will then log the containers out of the inventory tracking system and record the container number in the participant's chart.

- Participants will receive their home vaginal swab collections kit and Vaginal Symptom Diaries, along. Daily diaries will be used to record any vaginal bleeding, medications (oral or vaginal), placement of any vaginal products (e.g., lubricant), and sexual activity, as well as any side effects from study drugs. The swab kit will contain enough swabs to cover the collections that occur between randomization and the Week 4 visit. This includes daily swabbing for 7 days at weeks 0-1 and 3-4 (14 collections) and a weekly swab at Week 2 (1 collection). Vaginal Symptoms Diaries will be collected in conjunction with the swab collections. Kits will include sterile saline to apply to the swab prior to specimen collection, should the participant find vaginal swab dry and irritating. This technique minimizes participant discomfort and has been shown in MsFLASH pilot studies to not affect quality and quantity of specimens. Swabs will be mailed by participants directly to the study sites as they are collected.
- The Week 1 phone contact and Week 4 and 12 visits will be scheduled.
- Week 4 questionnaire(s) will be given. Participant instructions for swab collection, diaries and study medications will be provided. Women will be given written and verbal instructions to collect vaginal swab specimens on awakening in the morning, prior to placement of any intervention product within the vagina on prescribed days. The vaginal gel will be placed prior to going to bed at night, thus minimizing any effect on morning swab collection and reducing vaginal irritation. Women will be asked to complete daily vaginal diaries. Women will be asked to call the study hotline with any side effect concerns throughout the study, and inform the study team of any hospital admissions or potential serious adverse events. Women will be instructed on how to mail the self-collected swabs to the study site weekly.

8.3.3 Weeks 1, 3, 7, 11 Telephone Contacts

Telephone contacts will be conducted approximately 1, 3, 7, and 11 weeks after the randomization visit to review medication adherence and ascertain any unacceptable side effects of medication. The contacts at weeks 3 and 11 will primarily serve as a reminder of the upcoming visits and a reminder to complete daily swab collections for 7 days prior to the visits.

8.3.4 Week 4 Visit (V2)

Approximately 1-2 weeks before the Week 4 visit, participants will be called to:

- schedule or confirm their Week 4 visit;
- be reminded to complete their daily swab collections and vaginal symptoms diaries for the 7 days prior to their visit;
- asked to complete the Week 4 follow-up questionnaire (which includes many of the items collected at baseline, such as depressive symptoms, anxiety, sleep, and sexual function); and
- be reminded to bring their vaginal swabs, diaries, and study medication containers to the visit.

Required elements of the Week 4 visit:

- Review medication adherence by collecting containers and counting (tablets) or weighing (gel).
- Review swab collections and vaginal symptom diary completion adherence.
- Ascertain any unacceptable side effects of medication.
- Answer any questions regarding the medication, swab collection, or diaries.
- Review daily diaries of vaginal symptoms, bleeding and medication use collected during the first 4 weeks to confirm they are being completed accurately and completely.
- Review the Week 4 follow-up questionnaire for missing items. If the participant did not bring the questionnaire to the visit, she will be asked to complete it during the visit.

In addition, all measures collected at baseline will be repeated:

- A standardized pelvic exam with documentation of vulvar and vaginal skin breaks, discoloration, amount of lubrication and discomfort and collection of vaginal samples for yeast culture, vaginal and rectal samples for the biorepository, and swab and cytobrush sampling to assess GU atrophy and inflammation will be completed. Vaginal smears for Nugent score and Maturation Index will be made.
- A rectal swab will be collected by the clinician.
- A vaginal biopsy will be conducted on women who consented to the biopsy (Seattle site only).
- Wet mount and pH to assess for acute infection will be collected. If yeast infection, BV, or trichomonas are seen, the participant will be referred to her regular provider. If positive for yeast, staff informs participant that over the counter treatment is an option. Participants will remain in study, on study drug.
- Participants will receive additional vaginal swab collection supplies and vaginal symptom diaries, along with instructions on how and when they are to be used. The swab kit will contain enough swabs to cover the collections that occur between the Week 4 and Week 12 visits and sterile saline as needed. This includes weekly swabs during weeks 5-11 (6 collections) and daily swabbing for 7 days at weeks 11-12 (7 collections). Vaginal symptoms diaries will be collected in conjunction with the swab collections and mailed by participants to the study site on a weekly basis.
- The Week 12 visit will be scheduled.
- Provide with week 12 questionnaire and sample collection kit.

8.3.5 Week 12 Visit (V3)

Approximately 1-2 weeks before the Week 12 visit, participants will be called to:

- schedule or confirm their Week 12 visit;
- be reminded to complete their daily swab collections and vaginal symptoms diaries for the 7 days prior to their visit, and to mail or bring them to the study site.
- asked to complete the Week 12 questionnaire (which includes many of the items collected at baseline, such as depressive symptoms, anxiety, sleep, and sexual function); and
- be reminded to bring their study medication containers to the visit.

Required elements of the Week 12 visit:

- Review medication adherence by collecting containers and counting (tablets) or weighing (gel).
- Review swab collections and vaginal symptom diary completion adherence.
- Collect and review daily diaries of bleeding and medication use collected during the last 8 weeks to confirm they were completed accurately and completely.
- Review the Week 12 follow-up questionnaire for missing items.

In addition, all measures collected at baseline will be repeated:

- A non-fasting blood sample for serum estrogen metabolite concentrations will be collected.
- Wet mount and pH to assess for acute infection will be collected. (If yeast, BV or trichomonas are seen, the participant will be referred to her regular provider)
- A standardized pelvic exam with documentation of vulvar and vaginal skin breaks, discoloration, amount of lubrication and discomfort will be conducted and collection of vaginal and rectal samples for the biorepository, vaginal swab and cytobrush sampling to assess GU atrophy and inflammation will be completed. Vaginal smears for Nugent score and Maturation Index will be made.
- A rectal swab will be collected by the clinician.
- A vaginal biopsy will be done for women who have consented (Seattle site only).
- Participants will be informed that they will receive information about study findings and their treatment assignment by mail at the time when study findings are being published (after the end of the trial).

8.4 Early Termination

Women who ask to terminate the study participation early will be encouraged to complete a final set of study questionnaires.

8.5 Adherence and Retention

Retention of study participants and their adherence with the study protocol is a dominant focus after the participant is enrolled. Retention has several components: adherence (using study medications) and participation (collecting swabs, attending follow-up visits, accepting phone calls, completing questionnaires). Personal contacts, visits, and follow-up phone calls will be the cornerstone of adherence and retention efforts. The DCC will coordinate scripts and provide interviewer and staff training and guidelines for standardized contacts. Each site will implement its own local retention efforts to complement study-wide efforts.

At each treatment contact, research staff will assess and encourage compliance, including offering suggestions to increase compliance in those who are not taking the medication as recommended. Study medications will be collected and counted or weighed at the Week 4 and Week 12 visits to estimate adherence.

8.6 Participation Payments

As a recognition of the time and effort women contribute to the study, participants will be paid $50 for each biopsy, $25 upon completion of each study visit, and $75 at study completion (maximum compensation per Seattle participant is $300 and per Minnesota participant $150; $175-325 for women with infection at baseline - anticipated <5% of women). Parking for study visits will also be reimbursed.

9. Data Collection

The questionnaires and scales selected for the study were identified by the Network as appropriate for all MsFLASH protocols. See Table 3 (below) for details.

Table 3: Data Collection Schedule

	Screening	Intervention		
Contact	PS	W0	W4	W12
Outcome Measures				
Vaginal symptoms (VSI, MBS)[19]	X	X	X	X
Vaginal biologic samples		X	X	X
Vaginal biopsies		X	X	X
Vaginal swabs (self-collected)		X	X	X
Physical measures: height, weight		X		
Sexual Function (FSFI),[46-48] FSDS-R, Item 1[48,61]		X	X	X
Quality of Life (MENQOL)[49,50]		X	X	X
Treatment satisfaction (Likert)				X
Patient Benefit Evaluation (PBE)				X
Other Measures of Interest				
Daily Impact (DIVA)[60]		X		X
Anxiety (GAD-7)[62]		X		X
Depression (PHQ-8)[63]		X		X
Insomnia (ISI)[64]		X		X
Urinary symptoms: incontinence (ISI, 3IQ),[65,66] bladder pain and recurrent UTIs		X		X
Prebiotics and probiotics		X		X
Rectal swabs (biorepository)		X	X	X
Blood hormone assays (biorepository serum, plasma)		X		X
Safety/ eligibility measures				
Informed consent	X			
Medical history	X			
Menopause status	X			
Adverse events		X	X	X
Adherence			X	X
Other				

Demographics		X		

9.1 Primary Outcome

Most Bothersome Symptom (MBS) Scale[18,23]: Most bothersome symptom rates as 0=none to 3=severe, maximum score=3, measuring the most bothersome symptom of vaginal dryness, itching, irritation, soreness, or dyspareunia.

9.2 Secondary Outcomes

9.2.1 Subjective Measures

Several self-reported measures related to sexual function and menopausal quality of life will be collected at baseline, Week 4, and Week 12, including:

Vaginal Symptom Index (VSI): The composite Vaginal Symptom Index (VSI) is a secondary outcome measure for this trial. The VSI asks about vaginal dryness, itching, irritation, soreness or pain with sexual activity with each symptom rated for 0=none to 3 =most bothersome, and then averaged for a total score of 0-3

Menopause Specific Quality of Life (MENQOL)[49,50]: The presence and bother associated with 29-different menopausal symptoms will be assessed using the MENQOL.

Female Sexual Function Index (FSFI)[46-48]: The FSFI is a 19-item self-report questionnaire that assesses sexual functioning in women over the past 4 weeks (total score 2–36, higher scores indicates better functioning).

Female Sexual Distress Scale (FSDS-R)[48]: The FSDS is a 12-item self-report questionnaire that assesses distress related to sexual dysfunction in the prior month. A single question on bother will be used, item 1 as this item has been shown to screen for bother[61]. If a participant responds affirmatively, the full questionnaire will be administered at her clinic visit.

Treatment Satisfaction (week 12): Likert scale satisfaction and Patient Benefit Evaluation (PBE) “Overall, do you believe that you have experienced a meaningful benefit from the study medication?” (yes/no)

9.2.2 Objective Vaginal Measures

Several objective vaginal biologic measures will be collected including:

- Vaginal pH (<5; $\geq$5) at baseline, Week 4, and Week 12;
- Vaginal epithelial thickness (Seattle site only) at baseline, Week 4, and Week 12;
- Vaginal Maturation Index (VMI) (% parabasal, intermediate, and superficial cells) at baseline and Week 12

Clinic vaginal specimen collection (baseline, 4, 12-week visits): Women will undergo standardized pelvic exam, and collection of vaginal biologic samples to assess GU atrophy and inflammation. Notation will be made of discomfort on examination. A Dacron swab will be used to collect a mid-vaginal sidewall sample and smeared on a microscope slide, then stored in a slide case for Nugent scoring. A vaginal culture swab will be collected and sent to the appropriate lab at the site for yeast culture at V1 and V2. One posterior vaginal fornix swabs will be collected and stored at -80C. Protease-free buffer will be introduced with 5 cc syringe for vaginal lavage, then aspirated and kept in cooled rack until processed, supernatant frozen at -80C in 1.8 mL aliquots, and stored at -80C. Vaginal cells will be collected by cytobrush and spatula that will be gently brushed along both vaginal sidewalls and posterior vaginal fornix. The cytobrush is then placed into a cryovial with RNA Cellprotect reagent. RNA Cellprotect specimens will be left at 4C for 24 hours, and then stored at -80C. The vaginal cells collected with the spatula will be smeared on a slide, fixed with 95% ethanol for at least 10 minutes, then the slides stored for scoring the Maturation Index.

Vaginal biopsy procedure (baseline, 4, 12-week visits, Seattle site only): In women who consent to biopsies, 1cc of lidocaine will be injected into the vaginal sidewall. A 3-4 mm wide x 1mm deep tissue sample will be taken with mini-Tischler biopsy forceps. Pressure and silver nitrate will be used to stop any bleeding noted. Biopsies will be placed in formalin and processed for epithelial thickness and estrogen receptor subtype.

Vaginal swab specimen collection (Daily during baseline, 4, and 12-week visits; weekly during weeks 1-3, 5-10): At home, participants will perform self-collection of vaginal swabs and mail them to the study site weekly, or bring them to their visit at Weeks 4 and 12. The swabs will be stored at the site frozen, and shipped

to the Fredricks' lab on a monthly basis for analysis after the end of the trial. We and others have shown that swabs obtained for PCR studies of the human microbiota are stable when held at room temperature for up to two weeks and have validated use of mail transport of self-collected swabs for these assays. Swabs will be sent in the mail and frozen at -80C upon receipt until subjected to DNA extraction as previously described.[51]

All samples, forms, database and cryovials will be linked via a unique ID and 2-digit cryovial number to the participant's study ID and type of visit. Staff will be provided procedures for shipping biospecimens and will follow universal precautions for infectious disease control.

Objective vaginal measure evaluation:

Vaginal pH (from all 318 participants at 3 time points) will be obtained by the study clinician at the time of each pelvic examination from vaginal samples obtained at the upper one third of the vagina and recorded in the physical examination form. The pH paper will be protected from light and checked for expiration prior to use.

Vaginal epithelial thickness (from all participants who agree to vaginal biopsy at the Seattle site, at 3 time points) will be processed, and paraffin embedded at the University of Washington. H&E slides will be prepared and measures of the distance from the epithelial surface to the basal membrane will be taken at 3 representative points and measured, taking care that all slides are properly prepared without tangential cuts. Interpretation of vaginal epithelial thickness will be done in the Mitchell Lab, Boston MA.

Vaginal Maturation Indices (from all 318 participants at 2 time points) will be shipped from the DCC to the University of Washington Research Cytology Laboratory located at Harborview Medical Center in Seattle, Washington. Alcohol fixed direct smears will be stained with standard Papanicolau stain (hematoxylin, orange G and eosin). Light microscopic examinations of all stained cytology specimens will be performed by three certified clinical cytologists. Cytologic smears will be examined initially at low power (10x) to assess for overall quality and cellularity. Smears containing only red blood cells or no cells, displaying predominant inflammatory reaction, or air-drying artifact were categorized as inadequate for evaluation. The maturation index (MI) will be determined and expressed as the percentage of parabasal cells, intermediate cells and superficial cells. The MI of a non-estrogenized postmenopausal woman has a predominance of parabasal cells and is typically lacking in superficial cells. Superficial cells are recognized by the cytologist as large polygonal cells with abundant cytoplasm and a small condensed pyknotic nucleus. Cells from the intermediate and parabasal layers are smaller and have an open spherical nucleus. Inflammation is recognized as an increased number of white blood cells and bacteria and may be associated with a hypoestrogenic state and genitourinary symptoms.

Nugent score (from all 318 participants at 3 time points). Vaginal smears created at clinic visits will be shipped to the Fredricks Lab, where they will be Gram stained and scored according to Nugent's criteria.

9.2.3 Collection of additional samples for biorepository and pilot studies:

In addition, blood samples collected at baseline and week 12, as well as vaginal swabs, vaginal cytobrush, rectal swabs, cervicovaginal lavage and biopsies (in consenting women from the Seattle site only) collected at baseline, Week 4, and Week 12 will be submitted to the biorepository proposed as Aim 1.3.

Clinic Rectal swab collection procedure (baseline, 4 and 12-week visits): After the vaginal exam the clinician will collect a rectal swab by inserting a moistened swab 1-2 cm into the rectum, turning it 360 degrees and removing it, and placing it in a sterile cryovial.

Serum and plasma biorepository specimen collection (baseline, 12-week visits): Non-fasting blood for estrogen metabolites will be processed on site, aliquoted into 0.5 ml cryovials for serum and plasma, frozen, and shipped on dry ice to the central biorepository at the Fred Hutchinson Cancer Research Center.

9.3 MsFLASH Common Measures and Other Measures of Interest

Questions on the screening and baseline questionnaires assess age, race, ethnicity, marital status, employment status, socio-economic status, education, menopausal status, and number and type of comorbid conditions. Race and ethnicity questions follow NIH guidelines; questions on menopausal status are based on definitions from the Stages of Reproductive Aging as adapted for survey use. Height and weight will also be measured at baseline.

In addition, the following MsFLASH common measures are collected at baseline and Week 12:

Generalized Anxiety Disorder (GAD-7)[62]**:** The GAD-7 is a 7-item self-report questionnaire that assesses

anxiety symptoms.

Patient Health Questionnaire (PHQ-8) [63]**:** The PHQ-8 is a self-report questionnaire that assesses mood and depressive symptoms.

Insomnia Severity Index (ISI)[64]**:** The ISI is a 7-item scale that evaluates the severity of insomnia retrospectively over the past week. The scale is more specific to insomnia symptoms than the Pittsburgh scale, which focuses more broadly on overall sleep quality.

Other measures collected at baseline and Week 12 will include vaginal quality of life measures (DIVA) urinary symptoms and intake of probiotics and prebiotics. Vaginal quality of life measures will be assessed with the Day-to Day Impact of Vaginal Aging (DIVA)[60] questionnaire, 23 questions in 4 domains. Urinary incontinence will be assessed with the Questionnaire for female Incontinence Severity Index (ISI),[65] a 2-item questionnaire that assesses incontinence severity and the 3-Incontinence Questions Tool, (3IQ),[66] a 3-item questionnaire that distinguishes stress from urge continence. We will ask single questions about: bladder pain, recurrent urinary tract infections and loss of urine during sexual activity. Use of a panty liner or use of clothing protection from urinary incontinence will be captured in the daily vaginal symptom diary. We will ask single questions about probiotic and prebiotic intake.

9.4 Pilot Translational Studies

Each study will include analysis of biorepository specimens from 25 randomly selected women from each treatment arm (total 75 women). Microbiome, immunology and reproductive hormone analyses will include the following.

A) Microbiome studies to evaluate the role of the microbial community and its metabolic functions in postmenopausal vaginitis symptoms:

- Broad range 16S gene PCR with high throughput Illumina sequencing using MiSeq will characterize the microbial community in all daily swabs collected by women at week 0, 4, and 12, plus weekly swabs (30 swabs per woman). DNA on vaginal swabs will be extracted using methods optimized, perfected in the Fredricks lab[53]. This approach provides information on which bacteria are present and their relative abundances, but does not reveal the concentrations of bacteria. In addition, swabs that have not contacted a human surface will be processed in parallel to serve as sham digest (negative) controls and a subset of swabs from the same time point from clinician collected samples will be processed to assess fecal contamination. Results will be analyzed using an internal analysis pipeline that we developed at the FHCRC specifically for this purpose Chimeras will be detected and deleted. Sham digest controls will also be processed in parallel to exclude bacterial contamination of reagents. Rank abundance plots of bacterial representation will be generated from each sample. This approach has the advantage of detecting novel bacterial species and community profiles. This comprehensive analysis will allow intelligent selection of time points and number of samples to analyze in a definitive study to query changes in microbiota with study interventions.
- Metabolomic profiling of 3 samples from each woman (baseline, 4, 12 week visits) will assess the variability, diversity and dynamic range of vaginal metabolites in post-menopausal women, which will allow appropriate power calculations for larger studies. This study will provide functional data on microbial communities, linking host and microbial metabolic networks.
- Rectal specimens will undergo DNA extraction and amplification in a similar fashion to vaginal microbiome specimens and will allow characterization of differences between the vaginal and rectal microbiome

B) Postmenopausal vaginal mucosal immune response studies to optimize methodology, and inform directions for more comprehensive future studies:

- Vaginal cytobrush samples will be used to optimize methods for gene expression profiling using RNASeq. Microarray fails in 30-60% of samples due to low quantity or quality of RNA.[26,67]
- RNASeq using ultralow levels of nucleic acid may be an improvement, but limited data exist for vaginal tissues. The Broad Institute (MGH) has expertise optimizing methods for new tissue types, with low levels of RNA such as formalin fixed tissues. This platform will facilitate investigation of immune response pathways in the postmenopausal vagina. With 30ng/uL of RNA the Broad will guarantee 50 million paired-end reads for every sample, but can obtain sequences from lower concentrations of nucleic acid. The Broad will construct the library, perform alignment and will provide a DAM file for bioinformatics analysis by the team at the FHCRC.

- Meso Scale Discovery platform (greater sensitivity than Luminex in plasma samples) will be used for analysis of vaginal fluid cytokines to determine dynamic range and detection threshold, facilitating the selection of relevant soluble markers of inflammation and appropriate sample sizes for hypothesis-testing studies. The core laboratories at the Massachusetts General Hospital run by Dr. Patrick Sluss use this assay routinely, and will help adapt it for vaginal fluid.
- Immunohistochemistry (IHC) for markers of immune cells found in the vaginal mucosa (CD3, CD4, CD8, HLA-DR, CD1a, CD68, elastase) will assess the most prevalent cell populations using standard IHC methods optimized in the Mitchell Laboratory for these markers (Figure 3). The goal is to understand the role of resident immune cells in inflammation and postmenopausal vaginal symptoms to target future analyses.

Figure 3. Immunohistochemistry for CD3+ cells in premenopausal ectocervix

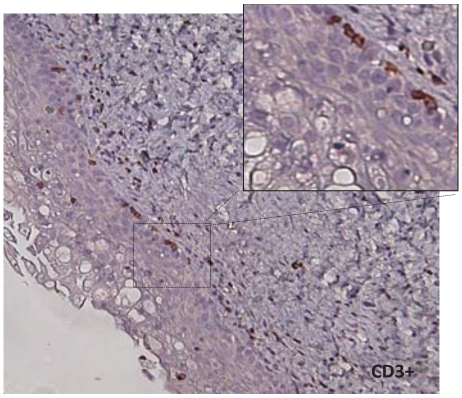

C) Studies of estrogen metabolite serum concentrations and ER presence in vaginal tissues to further understand the local response to circulating hormones and potential microbiome associations.

- Correlation of estrogen receptor expression (ERα or ERβ) measured by IHC with severity of symptoms and response to estrogen therapy will be determined.
- A large panel of estrogen metabolites will be measured using sensitive mass-spectrometry techniques and assessed for correlation with symptoms to determine whether the more extensive panel of hormone metabolites reveals unique associations with symptoms, or whether estradiol and estrone serum concentrations are sufficiently representative of the hormonal milieu and local vaginal hormonal concentrations and ER expression. Estrogens are currently thought to have a linear relationship with vaginal symptoms, but this conceptualization is likely oversimplified. Hormone metabolites and/or estrogen receptor status may play a role in modulating symptoms at a given estrogen level, thereby explaining the heterogeneity in women's symptoms and could potentially interact with the vaginal microbiome.

Estrogen receptor α and β IHC will be performed at the University of Washington Research Pathology Lab. Nuclear and cytoplasmic staining intensity of the basal, intermediate and superficial cell layers from vaginal biopsies will be quantified by protocols developed at the University of Washington (see photomicrographs of vaginal IHC ER staining in a postmenopausal and a premenopausal woman performed as pilot work in preparation for the trial, Figure 4 below). The staining will be compared between intervention groups. Staining will be assessed and correlated with vaginal pH and serum concentrations of estrone and estradiol.

Estrogen receptor IHC will be performed on the Bond III with the automated platform from Leica biosystems, using the polymer kit "Bond refine detection kit". Antibodies specific for ERα (clone SP1; Thermoscientific (Lab vision corporation), Rabbit monoclonal, Dusseldorf, Germany) will be used. Briefly, paraffin-embedded vaginal biopsies will be sectioned at 4 μm and mounted on slides. The slides will be deparaffinized with xylene, and rehydrated in decreasing concentrations of ethanol and deionized water and finally washed in a 1 x Tris-buffered saline solution (TBS; ph 7.4). Antigen retrieval will be performed by heating the tissue sections in EDTA buffer in a microwave for 20 minutes at 650 W. The primary antibody will be diluted to 1:50. ER B (clone 14C8, mouse monoclonal, Novus Biologicals) will be done similarly other than antigen retrieval is with EDTA for 15' and primary antibody dilution is 1: 100.

Premenopausal endometrial tissue will be used as a positive control. Staining intensity will be evaluated by 2 separate cytopathologists blinded to intervention group, pH and serum hormone concentrations with the following grading scale: 0 = no staining, 1 = faint, 2 = moderate, 3 = strong. Any disagreements will be adjudicated by a third blinded cytopathologist.

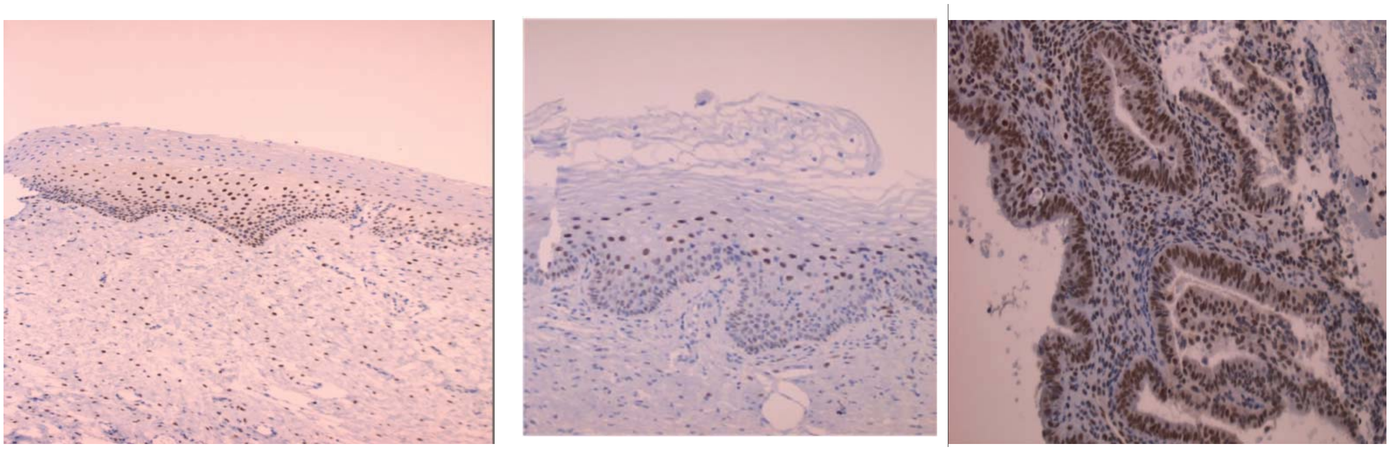

Figure 4. IHC pilot studies: vaginal tissue IHC staining of ERα protein in postmenopausal woman (left), premenopausal woman (center) and IHC staining of ERβ protein in perimenopausal endometrium (right).

Estradiol metabolite analyses will be performed using an ultrasensitive stable isotope dilution liquid chromatography/selected reaction monitoring/mass spectrometry (LC/SRM/MS) assay will be performed for estrogen metabolites. The limit of detection for each estrogen using 0.5 mL of serum is 0.156 pg/mL and linear standard curves are obtained up to 20 pg/mL. Specimens will be shipped from the DCC to a lab (to be determined) on dry ice according to MsFLASH specimen shipping and handling regulations. See example from MsFLASH03 pilot data on serum estrogen metabolite concentrations from single subject below (Figure 5).

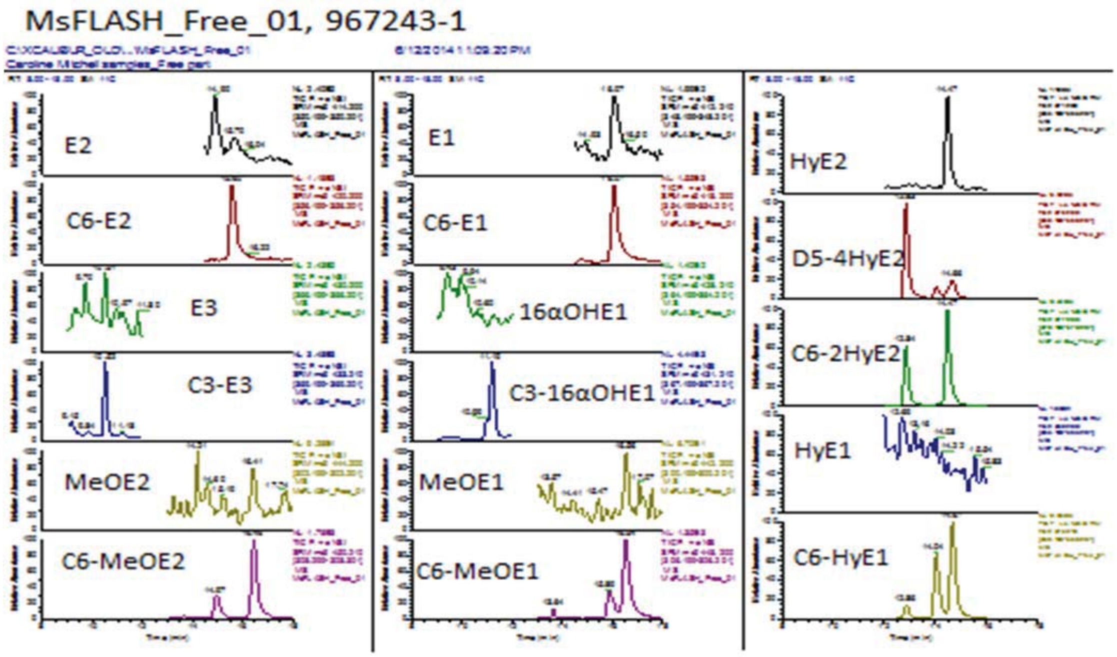

Figure 5. Ultrasensitive stable isotope dilution liquid chromatography/selected reaction monitoring/mass spectrometry (LC/SRM/MS) assay from single participant in MsFLASH03 trial

10. Statistical Considerations

10.1 Sample Size and Power

Women will be randomized in equal proportions to 3 treatment groups; 95 women per group provides 89% power to detect an effect size of 0.5 standard deviation (SD) units in change from baseline to week 12 in MBS severity between each intervention group and placebo,[19] based on a t-test with a 2-sided alpha of 2.5% to control for multiple testing. Enrollment of 318 subjects allows for 10% loss to follow-up. All randomized participants will contribute to analysis regardless of treatment adherence, according to the intention-to-treat principle.

10.2 Statistical Analysis

Treatment arm differences will be assessed by repeated measures linear regression models of the 4- and 12-week continuous outcome measures (MBS severity, VSI, dyspareunia, MENQOL, epithelial thickness, VMI as % parabasal cells) as a function of randomization assignment, baseline value of the outcome measure, and clinical site. Similarly, repeated measures logistic regression models will be applied to assess treatment differences in vaginal pH (< 5.0, $\geq$ 5.0) at follow-up. Robust standard errors will be estimated via generalized estimating equations (GEE) to adjust for correlation between repeated outcome measures. Differences in treatment satisfaction will be evaluated by linear regression models of the 12-week outcome as a function of randomization assignment and clinical site. For exploratory subgroup analysis, potential effect modifiers include: age, years post-menopause, VMI and pH at baseline. Additional analyses will assess the sensitivity of intervention effects to model adjustment for factors measured during the trial, including sexual activity and vaginal infection.

Missing Data: Every effort will be made to avoid missing data and to include all randomized subjects in the intent-to-treat analysis. If missingness is uncommon (<5%) and not differential across treatment arms, we will use a complete case analysis, which provides valid results under a missing completely at random assumption and hence is preferable to the last observation carried forward method.[68] If missing data are more common, or we see evidence of correlation with predictors of response in drop-out models, we propose to apply conditional mean multiple imputation[69] as it is valid under the somewhat less stringent assumption of missing at random, with sensitivity analyses to explore the impact of missing not at random.[70,71]

A subset of 75 participants will be studied to elucidate the pathophysiology of menopausal vaginitis, as described in Pilot Translational Studies above. Participants will be randomly selected from those who contribute vaginal biopsies, stratified by treatment arm. Laboratory assays will be performed by staff blinded to treatment arm and response. These analyses will be exploratory in nature.

Microbiome composition, diversity, and species richness will be measured using broad range 16S rRNA gene PCR with Illumina sequencing (MiSeq) and a classification pipeline developed by the Fredricks lab.[52-56] Microbiota will be quantified and per-sample species richness scores calculated. The association between taxon abundance and treatment response will be assessed using regression methods that address over-dispersion (e.g., hurdle or Dirichlet-multinomial mixture models[72]), in conjunction with lasso or other regularization to reduce dimensionality. The association between species richness and treatment group will use kernel regression methods to account for non-independence of counts implied by the hierarchy of phylogenetic relationships; one common metric is weighted UniFrac[73].We will use PICRUST[74] to investigate the functional differences in microbiomes across subjects as represented by inferred gene content and gene families. KEGG ontologies will be used to aggregate these genes into pathways to further elucidate differences in function, and gene set enrichment approaches will be used to determine if the representation of gene clusters or pathways differs between study groups. Metabolites detected in vaginal fluid by gas/liquid chromatography with mass spectroscopy will be assessed in each treatment group and differences between groups visualized using principle components analysis. Weighted correlation network analysis (WGCNA) will be used to identify groups of metabolites that show similar patterns of occurrence across samples. In addition, associations of metabolites with bacterial taxa and gene families or pathways noted above will be estimated using Spearman rank correlation or Procrustes analysis.

Vaginal inflammation will be measured using RNA extracted from vaginal cytobrush to compare epithelial gene expression. Online analysis tools such as Ingenuity and InnateDB will be used to assess genes and pathways associated with T-cell responses and adaptive immune response, compared to those associated with the mucosal innate immune response, before and after treatment. Meso Scale Discovery will be used to measure vaginal cytokines.

Vaginal estrogen receptors will be measured using IHC from vaginal biopsies to assess change in % vaginal cells per high power field expressing ERα and ERβ (scoring protocols refined in UW lab)[75,76]. Change in the ratio of ERβ to ERα will be calculated. Correlations with serum estradiol levels will be estimated.

11. Study Operations

11.1 Data Management

All study data will be entered into a centralized study database designed and maintained exclusively for MsFLASH network protocols by the DCC. Only de-identified data will be entered in the centralized database.

Data will be entered over a secure web-based application. Participant identifiers, such as name and contact information, will be maintained at each site for their participants, and accessible only to authorized staff at that site. All electronic data will be password-protected; participant files will be kept in locked cabinets.

The MsFLASH DCC will develop and implement systems for centralized data management and quality control. Network staff will use a secure website to access a web-based application for data entry and reporting activities. All MsFLASH data will reside in a central Oracle database maintained by the DCC. The staff at Network Sites will be granted access to only the data collected at their site. The DCC will be responsible for all database administration activities including database backups.

Access to all applications and databases will require user authentication. Database and web applications will have limits placed on idle session time. A limited number of invalid login attempts will be allowed before an account is temporarily locked. A password policy will enforce strong password selection criteria and periodic password changes. SSL encryption will be used to secure network traffic between browsers and the web server.

The DCC will have real-time access to all data entered by the Network Sites and will monitor recruitment and protocol progress. The DCC will extract data from all clinical sites for analysis and data sharing. Detailed data management procedures for Network Sites will be available in the Study Operations Manual.

11.2 Specimen Handling

Basic sample processing, shipping and handling information is shown in Table 4 below and details follow.

Table 4. Sample processing, shipping and handling

Sample	Week	Processing at site	# samples per participant each visit	Shipping
Blood (cryovials)	0, 12	Separation into serum, plasma	4	Ship to DCC on dry ice every 1 - 3 months
Vagina fluid smear for Nugent score	0, 4, 12	Smear on glass slide	1	Ship to Fredricks lab every 1 - 3 months
Vaginal fluid smear for VMI	0, 12	Smear on glass slide, fix at least 10 minutes with 95% ethanol, store dried slide	1	Ship to UW every 3 months
Vaginal swabs	0, 4, 12	Place in-80 c freezer	1	Ship to Fredricks lab on dry ice monthly
Yeast culture swab	0, 4	Send Culture Kit to Clinical lab<br>or inoculate agar plate, incubate and monitor for growth	1	Transport to local lab daily
Vaginal lavage<br>(2.0 mL cryovials)	0, 4, 12	Aliquot 1.8 mL lavage fluid into 3x 2.0 mL cryovails and freeze.-	5	Ship to MGH on dry ice every 1-3 months
Vaginal cytobrush	0, 4, 12	Cut off into a cryovial with 1mL RNAprotect Cell Reagent and freeze.	1	Ship to MGH on dry ice every 1-3 months
Vaginal biopsy (Seattle only)	0, 4, 12	Place in formalin	1	Transport to Alpers Lab for processing within 48 hours of collection
Rectal swab	0, 4, 12	Place in freezer	1	Ship to Fredricks lab on dry ice monthly
Self-collected vaginal swabs	Daily: Week 0-1, 3-4, 11-12<br>Weekly: weeks 2, 5-11	Place in freezer	1-7/week	Participants mail to site. Site sends to Fredricks Lab, on dry ice, monthly.

The DCC will provide sites with freezer-safe barcoded ID labels for all specimens to ensure that samples, forms, database and biospecimens (cryovials, vaginal swabs, slides and biopsies) are linked to the participant's study ID and type of visit via a unique ID and 2-digit biospecimen number. Staff will be trained and certified to collect the specimens and ship them according to MsFLASH protocols, using universal precautions for infectious disease control. Fixed vaginal biopsy specimens and VMI slides will be delivered directly to UW pathology. Pre-labeled Nugent slides and vaginal swabs collected in the clinic will be mailed or delivered directly to the Fredricks lab. Home-collected swabs will be sent by the participant to the site, and then the sites will ship these to the Fredricks Lab. Vaginal cytobrush samples and cervicovaginal lavage fluid will be shipped to the Mitchell Lab (MGH) by the sites. Neither the specimen repository nor the laboratories conducting the assays will have access to any participant identifiers. Specimens will be logged and tracked in the repository database, which will be accessible to all of the sites storing samples. The repository will provide regular inventory updates to the DCC specimen tracking system. The DCC will be responsible for lab pull requests and will coordinate with the repository to ensure the security and confidentiality of the specimens being shipped. All appropriate processing and shipping procedures will be outlined in the MsFLASH Study Operations Manual.

11.3 Packaging of Study Medications and Placebo

The DCC will coordinate the production, packaging, and distribution of study tablets and gel.

All study medications will be procured (active medications) or manufactured (placebo), packaged, and shipped by Sharp Clinical Services, Phoenixville, PA. They will be responsible for maintaining inventory; tracking expiration dates; conducting quality control (QC) measures; packaging and labeling the medications with unique container numbers; placing containers in cartons, as defined by the DCC's inventory control system for blinded medication tracking and dispensing; and shipping the cartons to study sites. Strict QC procedures at Sharp will involve multiple checks to ensure that the contents of the medication containers and cartons are consistent with their labels.

Containers of study tablets and gels will be distributed to participants by MsFLASH staff at each participating site. Upon receipt, medications shipped to each site will be logged into the local inventory by entering the carton label. To ensure that the correct medication is given to each participant, staff at the study sites will be required to enter both the container ID and the study participant's ID into the central database to complete the dispensation process.

Upon randomization, each participant will receive one container with the vaginal tablets and one containing the vaginal gel. Each container will contain enough medication to last throughout the 12-week treatment period. Site staff will count and collect remaining tablets and collect and weigh gel containers at the 4- and 12-week visits.

11.4 Quality Control

The overall system of quality assurance will be based on Good Clinical Practice (GCP) for assuring protection of human subjects, training on the protocol, data monitoring, laboratory monitoring, performance monitoring, and DCC quality assurance (QA) site visits.

11.4.1 GCP for Protection of Human Subjects

Documentation of Informed Consent. The original signed informed consent for each participant will be maintained in a secure locked area at each network site. A copy of the signed informed consent will be given to the participant. The receipt of signed informed consent for each participant for each trial will be data-entered in the central MsFLASH database.

IRB Review of Protocol. The FHCRC IRB will review the protocol as will each site's IRB where it will be implemented. Implementation will proceed upon approval from the FHCRC IRB and site-specific IRBs.

Privacy of Participant Information. Participant charts will be kept in secure locked areas and accessed only by approved staff and investigators.

Human Subjects Training and Security Training. All pertinent MsFLASH staff and investigators will have completed Human Subjects Training and Security Training and have documentation on file and available for review at site visits.

Standard Operating Procedures Manual. We will develop a standard operating procedure manual for both sites to use.

11.4.2 Training on the Protocol

Dr. Caroline Mitchell and the DCC clinic liaison will be responsible for site training with assistance from Dr. Reed as needed at the Seattle site. Core training elements will include 1) recruitment; 2) eligibility and screening; 3) informed consent; 4) enrollment and randomization; 5) blinding; 6) support for participant completion of the study diaries; 7) participant retention and adherence; 8) data collection and forms completion; 9) specimen collection, processing and shipping; 10) data entry and management; and 11) adverse event monitoring and reporting and participant safety. Training is required for all staff implementing the protocol. Staff certification is achieved by participating in 100% of the requisite trainings offered. Training will be offered through a combination of in-person and telephone methods. Centrally provided replacement staff training will be available as needed by conference call.

11.4.3 Data

Data quality will be assured by using standardized questionnaires, with automated range and validity checks at data entry. Data quality will be monitored by reports evaluating timeliness and completeness of data entry, within and between form consistency checks, and completeness of other required study tasks.

Data quality measures will be incorporated in the data management system to promote quality nearest the point of origin. The DCC will perform routine QA checks on the database. Results of QA reporting and any data issues arising from analysis activities will be submitted to the Network sites for resolution. Additional data quality checks will be conducted at the time of analyses, typically beginning with a visual examination of the data for outliers or other distributional abnormalities. Comparisons of distributions across sites and staff members and over time will be conducted for selected variables to examine potential sources of error or excess variability.

11.4.4 Laboratory Monitoring

Laboratory quality control will be assessed for all vaginal and blood sample analyses. There will be a 5% blinded duplicate analysis conducted. These 5% samples will receive blinded identification numbers and become the QC samples. Quality control for all laboratory work done within the Fredricks laboratory, the Mitchell laboratory and within the University of Washington laboratories is already established and follows strict guidelines, but will be reviewed by the DCC as part of the overall QC process during site visits and by electronic monitoring of study progress.

11.4.5 Performance Monitoring

Dr. Caroline Mitchell and the DCC clinic liaison will be responsible for performance monitoring with assistance from Dr. Reed as needed at the Seattle site. Network sites will be monitored for protocol performance in the following areas: 1) recruitment rates, 2) enrollment rates, 3) forms completion, 4) pill and gel dispensation records, 5) equipment maintenance documentation, 6) appropriate specimen collection and processing, 7) study site clinician performance of pelvic examination, wet mounts and vaginal specimen collection, 8) pelvic examination records and fidelity, 9) participant safety, and 10) upkeep of procedure manuals.

Ongoing support will occur via an email query system for questions from the sites and answers from the DCC, and monthly email updates from the DCC to the sites, including study progress and a question and answer digest. A telephone and email system for inquiring about protocol questions or problems will set up by the DCC.

11.5 Quality Assurance Site Visits

Site visits will be performed (2 visits per site) at each participating clinical site at the beginning of the study and approximately mid-way through recruitment. All site visits will assess whether adequate training has occurred, supplement the training if needed, and confirm that appropriate local documentation is in place for all protocol required procedures including eligibility evaluation, informed consent, data collection, entry and management, drug dispensing and tracking, specimen collection and processing, and participant safety.

Each site visit will also involve audits of a small random sample of participant charts. Site visit teams will include a member of the DCC and Dr. Mitchell who will oversee all study site clinician QA including pelvic examination, wet mounts, vaginal, rectal, and blood specimen handling and collection. The site visit will require the presence of the study site clinician, study coordinator, and additional clinical site expertise as needed. The DCC will work with the network sites to plan the agenda to best serve the project’s needs for problem-solving, monitoring, observation of tasks, as well as obtaining information regarding what additional database tools or

other resources are needed for efficient functioning at the sites.

Chart audits will be part of the site visits; charts will be audited for timeliness and accuracy of data entry. Performance monitoring will occur by recruitment reports, site visit observations, and adherence measures.

12. Study Monitoring

12.1 General

Progress in this study will be monitored in several ways, including reports on participant enrollment, adherence to follow-up procedures, and accrual of key study outcomes. The DCC will provide regular reports to the principal investigators (multi-PIs and the site PIs), as well as to the Data and Safety Monitoring Board (DSMB) and the National Institute on Aging (NIA). These reports will provide the basis for considerations of remedial actions or protocol changes and for considerations of directed publications and notifications to participants.

12.2 Accrual, Adherence, and Retention

A total of 318 women will be randomized to treatment (106 to each of the three arms). Seattle (Group Health Research Institute) and Minneapolis (University of Minnesota) are the clinical sites for this protocol. If needed, a third recruitment site will be added (Boston, Brigham and Women's).

Weekly reports of screening efforts will be collected from each participating site and reviewed by the DCC to ensure that sites are making progress toward study accrual. Adjustments will be made as needed at each clinical site along the way; for example, the number of targeted mailings sent out each week will be increased or decreased, depending on the number of interested and eligible women who respond.

Reports on accrual, intervention adherence, and completeness of participant follow-up activities, and other aspects of quality assurance will be reviewed regularly by the PI's to allow early identification of potential problems.

12.3 Safety Monitoring Plan (Adverse Events and Serious Adverse Events)

The eligibility criteria address participant safety; safety monitoring begins once a woman is randomized. Participants will also be asked to promptly call the study clinic and report any side effects that are distressing to them or if they are hospitalized. At each study follow-up contact, clinicians will record all reported adverse events, their severity (mild, moderate, severe) and duration. This includes the adverse events identified below, and any hospitalization, as well as an opportunity to identify "other" adverse events not listed. Reports of adverse and serious adverse events will be managed as indicated below.

An *adverse event (AE)* is defined as any untoward or unfavorable medical occurrence (including any abnormal sign, symptom, or disease) that occurs during the study (having been absent at baseline or if present at baseline, appears to worsen), regardless of its relationship to the study intervention.

The DCC will provide reports of adverse events to the DSMB. The DSMB will be provided with a summary of all adverse event data by unblinded treatment assignment for its review and recommendations.

A *serious adverse event (SAE)* is defined as any adverse event that:

- Results in death
- Is life threatening, or places the participant at immediate risk of death from the event as it occurred
- Requires or prolongs hospitalization
- Causes persistent or significant disability or incapacity
- Results in congenital anomalies or birth defects
- Is another condition which investigators judge to represent significant hazards

Serious adverse events will be managed medically or surgically, as appropriate, by the participant's primary care provider. If the participant is determined during a clinic contact to have an SAE for which she has not sought medical attention, she will be referred urgently to her provider. Study medication may or may not be stopped and an action plan will be determined at the discretion of the clinician. Protocol-mandated unblinding of the participant, her health care provider, or study staff is not required for any of these conditions because each should be managed as medically indicated regardless of treatment assignment (e.g., if stopping Vagifem®, Replens® or either placebo is medically indicated, then study medications should be stopped

without unblinding). The participant's treatment assignment will be provided to the local clinician and/or personal physician/health care provider, if requested as medically necessary.

The clinic site principal investigator will be responsible for ensuring that serious adverse events (either by participant or clinician report, or assessed during a clinic contact) are immediately reported (within 24 hours) to the IRB (according to local institutional requirements) and the DCC, which will promptly inform the NIA and the MsFLASH DSMB. The clinic site will then obtain additional, detailed information on the SAE on the *SAE Report Form*, which will then be forwarded to the IRB and DCC (and then to the NIA and DSMB). Additional information about and/or management of the SAE may be identified by the IRB or DSMB, and should be carried out promptly by the clinic site.

Symptom severity will be assessed on a scale of mild, moderate, severe or life-threatening.

Attribution, that is the likelihood that any specific adverse or a serious adverse event might be reasonably thought to be the result of a given study procedure will be scaled using the following five criteria: definitely, probably, possibly, probably not, and definitely not.

12.4 Vagifem® Safety and Potential Risks

Vagifem® 10 mcg - No serious adverse events from the use of new FDA approved ultra-low-dose estradiol vaginal tablet formulations (10 mcg) (indication for the treatment of atrophic vaginitis due to menopause) have been observed in trials. Vagifem® 10 mcg has minimal systemic absorption, is easy to use, and may be an option for women with prior breast cancer[77-80]. Treatment emergent adverse events reported at a frequency >=5%, for placebo and active drug respectively, were: back pain 2%,7%; diarrhea 0%, 5%; vulvovaginal mycotic infection 3%, 8%; vulvo-vaginal pruritus 2%, 8% (placebo n=103, Vagifem® 10mcg =205).

Vagifem® is only used in the vagina but risks of oral higher dose formulations must be considered. Observational and trial data for oral hormonal products at doses 100 times greater than the local vaginal estrogen dose proposed in this trial have shown that women taking standard dose oral ET alone, for 5 years or less, appear to only have a slight increased risk for VTE and stroke that increases with age.[81] There is strong evidence to suggest that low dose and transdermal ET may be associated with even lower overall risks of VTE[81-85] and cardiovascular disease[86,87] than standard dose and oral ET. Average serum concentrations of estradiol and estrone are shown in Table 5 to the right.

Table 5: Serum Concentrations (pg/mL)

	Estradiol	Estrone	Estrone Sulfate
Day 1	10.09	20.22	214.9
Day 14	7.35	20.67	263.5
Day 83	5.5	17.13	158.5

Any women with vaginal bleeding in the prior 2 years or with a history of abnormal bleeding that was not evaluated and treated will not be randomized. Any bleeding that occurs during the trial will be referred to the patient's provider as women will be carefully screened for endometrial neoplasia at baseline. There is no data to suggest that exposure to Vagifem® 10mcg daily for 2 weeks, then twice weekly for 10 weeks increases the risk of breast or endometrial carcinoma.

12.5 Replens® Safety and Potential Risks

No serious adverse events from the use of Replens® have been observed. Potential increased vaginal discharge and odor[88] have been described.

12.6 Placebo Vaginal Tablet and Gel Safety and Potential Risks

The placebo tablet coating and excipient ingredients will be the same as are used for Vagifem®. The coating will be hydroxypropyl methylcellulose (hypromellose) and polyethylene glycol. The excipients will be hypromellose, lactose monohydrate, maize starch, and magnesium stearate. Placebo vaginal gel (hydroxyethylcellulose gel) will be an inert nonallergenic sterile formulation without moisturizing properties (non-bioadhesive) without carbophol or polycarbophol. Hydroxyethylcellulose gel was chosen due to its safety profile and favorable experience in prior vaginal microbicide trials.[43,44]

12.7 Adverse Event Monitoring and Management Related to Study Drugs

Vagifem®, Replens®, and placebo gel will be monitored for expiration (shelf life) such that no drug will be dispensed that will expire prior to participant completion.

Self-management strategies for mild to moderate bothersome symptoms potentially related to Vagifem®, Replens®, or vaginal placebo gel (hydroxyethylcellulose gel) are appropriate and may include use of a non-allergenic panty liner. Use of panty liners will be noted in the daily diary.

Suggested strategies will be provided in a handout that is given to participants at the time study pills and gel are dispensed. Participants will be referred to their primary care physicians for management of severe/intolerable symptoms that do not respond to self-management strategies. If the participant identifies symptoms as continuing despite management efforts, study pills or gel will be discontinued.

12.8 Vaginal Specimen Collection Safety and Potential Risks

Vaginal infections: At baseline, strict screening methods for vaginal infection and exclusion criteria will be followed. Women with vaginal infections at baseline identified by wet mount will not be randomized until fully treated and test of cure documented by staff. There is a small increased risk for yeast infection with Vagifem®. AEs will be monitored. Participants will receive phone calls from study staff at weeks 1, 3, 7, and 11. In addition, all women will be encouraged to contact study staff with any potential AEs. Women will be asked to call the study hotline with any side effect concerns throughout the study, and inform the study team of any hospital admissions or potential serious adverse events. Women who develop vaginal infections during the trial will be referred to their primary care provider (wet mounts performed at weeks 0 and 4). If yeast infection is diagnosed, staff will also inform participant that over the counter treatment is an alternative.

Self-collection of vaginal swabs: Careful instructions on how to collect vaginal swab specimens (both verbal and written with pictorial depiction as piloted in MsFLASH03) will be provided, thus minimizing vaginal irritation or discomfort. Women will be provided with sterile saline to apply to the swabs should they prove dry and irritating on placement. This method was piloted in the EVA MsFLASH03 study and proved effective without affecting specimen quality.

Speculum placement can be uncomfortable in postmenopausal women and can even cause minor skin abrasions and bleeding. Training to minimize tissue trauma during vaginal specimen collection will occur prior to start up and tracking and monitoring will continue at each site under the direction of the site PIs. All AEs related to vaginal specimen collection will be tracked and monitored at each site and reported to the DSMB as required.

Vaginal biopsies (performed at Seattle site only) can result in discomfort, anxiety, bleeding, and theoretically, infection. Only trained clinicians will be allowed to obtain biopsy samples. Safety measures to ensure minimal burden to participants include:

- Blood pressure and heart rate of all participants that consent to biopsy will be obtained prior to the procedure and repeated if participant expresses light headedness or dizziness during or after the procedure. This is highly unlikely, but can occur in anxious individuals or due to minor pain. See Appendix A for criteria on management of heart rate and blood pressure outside of normal ranges and the classification of normal ranges.
- Light bleeding or spotting following the procedure is anticipated and is managed with silver nitrate. Participants will be provided with panty liners.
- Pain will be minimized with the use of topical lidocaine. Participants will be queried about potential allergic reactions to lidocaine prior to the procedure. Some women may elect to have a vaginal biopsy without medications.
- Risk of infection is remote, but should this occur, the participant will be referred to her primary care provider for management. Sterile technique will be maintained throughout the procedure.

One week following biopsy, all women will be called by the research assistant to query regarding symptoms. If there is concern regarding pain or bleeding, the participant will be re-evaluated in the research clinic by the study interventionist or site physician. If there is concern for an infection following vaginal biopsy, the participant will be referred to her primary care provider. Participants will be provided with clinic site number to contact the study clinician should they have a concern prior to the one-week post procedure phone call. All AEs related to vaginal biopsies will be tracked and monitored at each site and reported to the DSMB as required.

<u>12.9 Venipuncture </u>Safety and Potential Risks:

Venipuncture is associated with transient pain, and on occasion bruising, at the venipuncture site which may persist for 1–2 weeks. Only certified phlebotomists, nurses or physicians will be allowed to obtain blood samples.

12.10 Other Safety Issues

<u>Questionnaires</u>: Though unlikely, answering questions about vaginal and urinary symptoms, sexual function and mood may cause psychological discomfort in some subjects. However, the questions asked are not substantially different from questions routinely used by clinicians in assessing patients with vaginal, urinary, and sexual or mood symptoms. Other questions might cause embarrassment or discomfort, depending on the individual and the circumstance.

12.11 Trial Monitoring (DSMB)

An independent Data and Safety Monitoring Board (DSMB) will be established by the National Institute on Aging (NIA), the sponsor of the study, to review study activities and data to ensure data quality and participant safety. The protocol and consent forms will be approved by the DSMB before the trial begins. All subsequent changes to the protocol will be provided and any changes to the protocol with potential impact on safety or ethical conduct of the trial will require DSMB approval before implementation. The DCC will work with the DSMB to develop the trial monitoring procedures. During the study, the DCC will provide the DSMB with routine reports on recruitment, timeliness and quality of data collection, adherence to protocol, and adverse effects as described in the protocol, including unblinded treatment comparisons for each of their regular meetings. Serious adverse effects will be reported to the DSMB within 48 hours of DCC notification.

13. Study Timeline

Participant recruitment is planned for 6 months, with a 3 month intervention period. The overall timeline for the study is shown in *Table 5.*

Table 6: Projected Timeline

**Projected Timeline**	**Y 1**		**Y 2**		**Y 3**		**Y 4**		**Y5**	
Implementation planning										
**Aim 1:** Recruit randomize and follow RCT (n=318**)**										
**Aim 1.1 – 1.3:** Laboratory Work and create biorepository										
Analysis/publication of Aims 1, 1.1, 1.2 trial data										
**Aim 1.4:** Vaginal Microbiome, Inflammation, Hormone assays (n=75)										
Analysis/publication Aim 1.4 data, maintain biorepository										

14. Summary of the MsFLASH Network

In 2005, the NIH convened a State-of-the-Science conference on Management of Menopause-related Symptoms. Recognizing the need to accelerate the pace at which new interventions for relief of VMS are tested, the NIA released RFA-AG-08-004 entitled, “New Intervention for Menopausal Symptoms (U01)”. The MsFLASH Network (Menopause Strategies: Finding Lasting Answers for Symptoms and Health) was selected for this award. The network was comprised of 5 Clinical Sites and a Data Coordinating Center, each led by Principal Investigators who are experienced clinical trialists and expert women’s health researchers. The clinical sites were chosen to optimize the network with respect to: 1) expertise in VMS physiology, epidemiology, and management; 2) proven experience executing randomized clinical trials on VMS; 3) multidisciplinary perspectives; 4) coverage of a broad range of candidate VMS interventions including CAM and behavioral strategies; 5) access to diverse populations of mid-life women including special populations; 6) regional diversity; and 7) the strong desire to work together effectively to accomplish the network mission.

During the first grant period, the MsFLASH network worked to fulfill the main objective of RFA-AG-08-004

which was to, “…design and conduct multiple concurrent clinical intervention studies accommodating a wide scope of populations and intervention strategies” with the long term goal of finding new ways “to alleviate the most common, bothersome symptoms of the menopausal transition.” [RFA, pages 3-4]. Under this overarching goal, the MsFLASH network conducted four randomized controlled trials testing a range of behavioral, mind-body, hormonal and pharmacologic interventions to treat hot flashes and other menopausal symptoms.

During this phase (a 5-year funding period), we will: 1) conduct a large multicenter trial on a relatively understudied are of menopause - vaginal health and sexual function with important biologic endpoints (Aim 1), and 2) based on review of all available evidence including the MsFLASH Trials, develop and pilot-test a comprehensive, state-of-the-art, multi-media national resource to translate and disseminate tailored information and to facilitate informed decision-making for managing menopausal symptoms (Aim 2). Our *long-term goal* is to improve the quality of life and health of aging, midlife women.

Drs. Guthrie (FHCRC), LaCroix (UCSD), and Reed (UW) are Multiple-Principal Investigators with responsibility for leadership and oversight of all network activities during this project period (2015-2020). Dr. Reed and Dr. Mitchell will be responsible for the oversight of the protocol, quality assurance and safety monitoring of the Vaginal Health Trial (Aim 1). Drs. Ensrud and Diem (Minneapolis) and Reed and Newton (Seattle, WA) will lead the clinical sites. and Dr. Mitchell (Boston, MA) stands ready to lead a clinical site as needed to meet recruitment goals. Drs. Fredricks (FHCRC), Mitchell (MGH) and Garcia (UW) will provide leadership on the MsFLASH biorepository and laboratory studies for Aim 1. Dr. LaCroix and Dr. Newton will be leading efforts for Aim 2 (not part of this protocol).

15. REFERENCES

1. NAMS. The role of local vaginal estrogen for treatment of vaginal atrophy in postmenopausal women: 2007 position statement of The North American Menopause Society. Menopause 2007;14:355-69.

2. Kingsberg SA, Krychman ML. Resistance and Barriers to Local Estrogen Therapy in Women with Atrophic Vaginitis. J Sex Med 2013;10:1567-74.

3. Stiles M, Redmer J, Paddock E, Schrager S. Gynecologic issues in geriatric women. J Womens Health 2012;21:4-9.

4. Nappi RE, Kokot-Kierepa M. Women's voices in the menopause: results from an international survey on vaginal atrophy. Maturitas 2010;67:233-8.

5. Santoro N, Komi J. Prevalence and impact of vaginal symptoms among postmenopausal women. J Sex Med 2009;6:2133-42.

6. Sturdee DW, Panay N. Recommendations for the management of postmenopausal vaginal atrophy. Climacteric 2010;13:509-22.

7. Levine KB, Williams RE, Hartmann KE. Vulvovaginal atrophy is strongly associated with female sexual dysfunction among sexually active postmenopausal women. Menopause 2008;15:661-6.

8. Wines N, Willsteed E. Menopause and the skin. Australas J Dermatol 2001;42:149-8.

9. Pastore LM, Carter RA, Hulka BS, Wells E. Self-reported urogenital symptoms in postmenopausal women: Women's Health Initiative. Maturitas 2004;49:292-303.

10. Greendale GA, Zibecchi L, Petersen L, Ouslander JG, Kahn B, Ganz PA. Development and validation of a physical examination scale to assess vaginal atrophy and inflammation. Climacteric 1999;2:197-204.

11. Cardozo L, Bachmann G, McClish D, Fonda D, Birgerson L. Meta-analysis of estrogen therapy in the management of urogenital atrophy in postmenopausal women: second report of the Hormones and Urogenital Therapy Committee. Obstet Gynecol 1998;92:722-7.

12. Suckling J, Lethaby A, Kennedy R. Local oestrogen for vaginal atrophy in postmenopausal women. Cochrane Database Syst Rev 2006;CD001500.

13. Shulman LP, Portman DJ, Lee WC, Balu S, Joshi AV, Cobden D, Wang Q, Pashos CL. A retrospective managed care claims data analysis of medication adherence to vaginal estrogen therapy: implications for clinical practice. J Womens Health (Larchmt) 2008;17:569-78.

14. Manonai J, Theppisai U, Suthutvoravut S, Udomsubpayakul U, Chittacharoen A. The effect of estradiol vaginal tablet and conjugated estrogen cream on urogenital symptoms in postmenopausal women: a comparative study. J Obstet Gynaecol Res 2001;27:255-60.

15. ACOG Practice Bulletin. ACOG Practice Bulletin No. 119: Female sexual dysfunction. Obstet Gynecol 2011;117:996-1007.

16. Patient information. Treating menopausal symptoms. Am Fam Physician 2010;82:809-10.

17. Dezzutti CS, Brown ER, Moncla B, Russo J, Cost M, Wang L, Uranker K, Kunjara Na Ayudhya RP, Pryke K, Pickett J, Leblanc MA, Rohan LC. Is wetter better? An evaluation of over-the-counter personal lubricants for safety and anti-HIV-1 activity. PLoS One 2012;7:e48328. PMCID: PMC3492332

18. US Food and Drug Administration. Guidance for industry. Estrogen and estrogen/progestin drug products to treat vasomotor symptoms and vulvar and vaginal atrophy symptoms–Recommendations for clinical evaluation. Draft guidance US Food and Drug Administration 2003;

19. Bachmann G, Lobo RA, Gut R, Nachtigall L, Notelovitz M. Efficacy of low-dose estradiol vaginal tablets in the treatment of atrophic vaginitis: a randomized controlled trial. Obstet Gynecol 2008;111:67-76.

20. Bachmann GA, Komi JO. Ospemifene effectively treats vulvovaginal atrophy in postmenopausal women: results from a pivotal phase 3 study. Menopause 2010;17:480-6.

21. Labrie F, Cusan L, Gomez JL, Cote I, Berube R, Belanger P, Martel C, Labrie C. Effect of one-week treatment with vaginal estrogen preparations on serum estrogen levels in postmenopausal women. Menopause 2009;16:30-6.

22. Freedman M, Kaunitz AM, Reape KZ, Hait H, Shu H. Twice-weekly synthetic conjugated estrogens vaginal cream for the treatment of vaginal atrophy. Menopause 2009;16:735-41.

23. Bachmann G, Bouchard C, Hoppe D, Ranganath R, Altomare C, Vieweg A, Graepel J, Helzner E. Efficacy and safety of low-dose regimens of conjugated estrogens cream administered vaginally. Menopause 2009;16:719-27.

24. Simon J, Nachtigall L, Gut R, Lang E, Archer DF, Utian W. Effective treatment of vaginal atrophy with an ultra-low-dose estradiol vaginal tablet. Obstet Gynecol 2008;112:1053-60.

25. Marrazzo JM, Thomas KK, Fiedler TL, Ringwood K, Fredricks DN. Risks for acquisition of bacterial vaginosis among women who report sex with women: a cohort study. PLoS One 2010;5:e11139. PMCID: PMC2886123

26. Hummelen R, Macklaim JM, Bisanz JE, Hammond JA, McMillan A, Vongsa R, Koenig D, Gloor GB, Reid G. Vaginal microbiome and epithelial gene array in post-menopausal women with moderate to severe dryness. PLoS One 2011;6:e26602. PMCID: PMC3206802

27. Nelson DB, Bellamy S, Odibo A, Nachamkin I, Ness RB, Allen-Taylor L. Vaginal symptoms and bacterial vaginosis (BV): how useful is self-report? Development of a screening tool for predicting BV status. Epidemiol Infect 2007;135:1369-75. PMCID: PMC2870698

28. Ichinohe T, Pang IK, Kumamoto Y, Peaper DR, Ho JH, Murray TS, Iwasaki A. Microbiota regulates immune defense against respiratory tract influenza A virus infection. Proc Natl Acad Sci U S A 2011;108:5354-9. PMCID: PMC3069176

29. Turnbaugh PJ, Hamady M, Yatsunenko T, Cantarel BL, Duncan A, Ley RE, Sogin ML, Jones WJ, Roe BA, Affourtit JP, Egholm M, Henrissat B, Heath AC, Knight R, Gordon JI. A core gut microbiome in obese and lean twins. Nature 2009;457:480-4. PMCID: PMC2677729

30. Turnbaugh PJ, Ley RE, Hamady M, Fraser-Liggett CM, Knight R, Gordon JI. The human microbiome project. Nature 2007;449:804-10.

31. Gonzalez A, Clemente JC, Shade A, Metcalf JL, Song S, Prithiviraj B, Palmer BE, Knight R. Our microbial selves: what ecology can teach us. EMBO Rep 2011;12:775-84. PMCID: PMC3147266

32. Martin HL, Richardson BA, Nyange PM, Lavreys L, Hillier SL, Chohan B, Mandaliya K, Ndinya-Achola JO, Bwayo J, Kreiss J. Vaginal lactobacilli, microbial flora, and risk of human immunodeficiency virus type 1 and sexually transmitted disease acquisition. J Infect Dis 1999;180:1863-8.

33. Hillier SL, Krohn MA, Klebanoff SJ, Eschenbach DA. The relationship of hydrogen peroxide-producing lactobacilli to bacterial vaginosis and genital microflora in pregnant women. Obstet Gynecol 1992;79:369-73.

34. Wilks M, Wiggins R, Whiley A, Hennessy E, Warwick S, Porter H, Corfield A, Millar M. Identification and H(2)O(2) production of vaginal lactobacilli from pregnant women at high risk of preterm birth and relation with outcome. J Clin Microbiol 2004;42:713-7. PMCID: PMC344438

35. Yudin MH, Landers DV, Meyn L, Hillier SL. Clinical and cervical cytokine response to treatment with oral or vaginal metronidazole for bacterial vaginosis during pregnancy: a randomized trial. Obstet Gynecol 2003;102:527-34.

36. Pabich WL, Fihn SD, Stamm WE, Scholes D, Boyko EJ, Gupta K. Prevalence and determinants of vaginal flora alterations in postmenopausal women. J Infect Dis 2003;188:1054-8.

37. Heinemann C, Reid G. Vaginal microbial diversity among postmenopausal women with and without hormone replacement therapy. Can J Microbiol 2005;51:777-81.

38. Cauci S, Driussi S, De SD, Penacchioni P, Iannicelli T, Lanzafame P, De SF, Quadrifoglio F, de AD, Guaschino S. Prevalence of bacterial vaginosis and vaginal flora changes in peri- and postmenopausal women. J Clin Microbiol 2002;40:2147-52. PMCID: PMC130764

39. Flores R, Shi J, Fuhrman B, Xu X, Veenstra TD, Gail MH, Gajer P, Ravel J, Goedert JJ. Fecal microbial determinants of fecal and systemic estrogens and estrogen metabolites: a cross-sectional study. J Transl Med 2012;10:253. PMCID: PMC3552825

40. Petricevic L, Kaufmann U, Domig KJ, Kraler M, Marschalek J, Kneifel W, Kiss H. Rectal Lactobacillus species and their influence on the vaginal microflora: a model of male-to-female transsexual women. J Sex Med 2014;11:2738-43.

41. Grady D. Clinical practice. Management of menopausal symptoms. N Engl J Med 2006;355:2338-47.

42. van der Laak JA, de Bie LM, de LH, de Wilde PC, Hanselaar AG. The effect of Replens on vaginal cytology in the treatment of postmenopausal atrophy: cytomorphology versus computerised cytometry. J Clin Pathol 2002;55:446-51. PMCID: PMC1769660

43. Richardson BA, Kelly C, Ramjee G, Fleming T, Makanani B, Roberts S, Musara P, Mkandawire N, Moench T, Coletti A, Soto-Torres L, Karim SA. Appropriateness of hydroxyethylcellulose gel as a placebo control in vaginal microbicide trials: a comparison of the two control arms of HPTN 035. J Acquir Immune Defic Syndr 2013;63:120-5. PMCID: PMC3625489

44. Mayer KH, Peipert J, Fleming T, Fullem A, Moench T, Cu-Uvin S, Bentley M, Chesney M, Rosenberg Z. Safety and tolerability of BufferGel, a novel vaginal microbicide, in women in the United States. Clin Infect Dis 2001;32:476-82.

45. Ekin M, Yasar L, Savan K, Temur M, Uhri M, Gencer I, Kivanc E. The comparison of hyaluronic acid vaginal tablets with estradiol vaginal tablets in the treatment of atrophic vaginitis: a randomized controlled trial. Arch Gynecol Obstet 2011;283:539-43.

46. Rosen R, Brown C, Heiman J, Leiblum S, Meston C, Shabsigh R, Ferguson D, D'Agostino R, Jr. The Female Sexual Function Index (FSFI): a multidimensional self-report instrument for the assessment of female sexual function. J Sex Marital Ther 2000;26:191-208.

47. Wiegel M, Meston C, Rosen R. The female sexual function index (FSFI): cross-validation and development of clinical cutoff scores. J Sex Marital Ther 2005;31:1-20.

48. Derogatis L, Clayton A, Lewis-D'Agostino D, Wunderlich G, Fu Y. Validation of the female sexual distress scale-revised for assessing distress in women with hypoactive sexual desire disorder. J Sex Med 2008;5:357-64.

49. Hilditch JR, Lewis J, Peter A, van MB, Ross A, Franssen E, Guyatt GH, Norton PG, Dunn E. A menopause-specific quality of life questionnaire: development and psychometric properties. Maturitas 1996;24:161-75.

50. Lewis JE, Hilditch JR, Wong CJ. Further psychometric property development of the Menopause-Specific Quality of Life questionnaire and development of a modified version, MENQOL-Intervention questionnaire. Maturitas 2005;50:209-21.

51. Nugent RP, Krohn MA, Hillier SL. Reliability of diagnosing bacterial vaginosis is improved by a standardized method of gram stain interpretation. J Clin Microbiol 1991;29:297-301. PMCID: PMC269757

52. Hamady M, Lozupone C, Knight R. Fast UniFrac: facilitating high-throughput phylogenetic analyses of microbial communities including analysis of pyrosequencing and PhyloChip data. ISME J 2010;4:17-27. PMCID: PMC2797552

53. Srinivasan S, Liu C, Mitchell CM, Fiedler TL, Thomas KK, Agnew KJ, Marrazzo JM, Fredricks DN. Temporal variability of human vaginal bacteria and relationship with bacterial vaginosis. PLoS One 2010;5:e10197. PMCID: PMC2855365

54. Fredricks DN, Fiedler TL, Thomas KK, Mitchell CM, Marrazzo JM. Changes in vaginal bacterial concentrations with intravaginal metronidazole therapy for bacterial vaginosis as assessed by quantitative PCR. J Clin Microbiol 2009;47:721-6. PMCID: PMC2650913

55. Hamady M, Walker JJ, Harris JK, Gold NJ, Knight R. Error-correcting barcoded primers for pyrosequencing hundreds of samples in multiplex. Nat Methods 2008;5:235-7.

56. Lauber CL, Zhou N, Gordon JI, Knight R, Fierer N. Effect of storage conditions on the assessment of bacterial community structure in soil and human-associated samples. FEMS Microbiol Lett 2010;307:80-6. PMCID: PMC3148093

57. Mitchell C, Balkus J, Agnew K, Lawler R, Hitti J. Changes in the vaginal microenvironment with metronidazole treatment for bacterial vaginosis in early pregnancy. J Womens Health (Larchmt) 2009;18:1817-24. PMCID: PMC2864467

58. Mitchell CM, Balkus J, Agnew KJ, Cohn S, Luque A, Lawler R, Coombs RW, Hitti JE. Bacterial vaginosis, not HIV, is primarily responsible for increased vaginal concentrations of proinflammatory cytokines. AIDS Res Hum Retroviruses 2008;24:667-71.

59. Mitchell C, Hitti J, Paul K, Agnew K, Cohn SE, Luque AE, Coombs R. Cervicovaginal shedding of HIV type 1 is related to genital tract inflammation independent of changes in vaginal microbiota. AIDS Res Hum Retroviruses 2011;27:35-9. PMCID: PMC3034096

60. Huang AJ, Gregorich SE, Kuppermann M, Nakagawa S, Van Den Eeden SK, Brown JS, Richter HE, Walter LC, Thom D, Stewart AL. Day-to-Day Impact of Vaginal Aging questionnaire: a multidimensional measure of the impact of vaginal symptoms on functioning and well-being in postmenopausal women. Menopause. 2015 Feb;22(2):144-54.

61. Carpenter JS, Reed SD, Guthrie KA, Larson JC, Newton KM, Lau RJ, Learman LA, Shifren JL. Using an FSDS-R Item to Screen for Sexually Related Distress: A MsFLASH Analysis. Sex Med 2015;3:7-13. PMCID: PMC4380908

62. Spitzer RL, Kroenke K, Williams JB, Lowe B. A brief measure for assessing generalized anxiety disorder: the GAD-7. Arch Intern Med 2006;166:1092-7.

63. Gilbody S, Richards D, Brealey S, Hewitt C. Screening for depression in medical settings with the Patient Health Questionnaire (PHQ): a diagnostic meta-analysis. J Gen Intern Med 2007;22:1596-602. PMCID: PMC2219806

64. Smith MT, Wegener ST. Measures of sleep: The Insomnia Severity Index, Medical Outcomes Study (MOS) Sleep Scale, Pittsburgh Sleep Diary (PSD), and Pittsburgh Sleep Quality Index (PSQI). Arth Care Res 2003;49:S184-S196.

65. Sandvik H. Validity of the incontinence severity index: comparison with pad-weighing tests. International Urogynecology Journal, October 2006, Volume 17, Issue 5, pp 520-524.

66. Lin KW. Brief Questions Accurately Diagnose Urinary Incontinence. Am Fam Physician. 2006;74(6):1027-1028.

67. Dahn A, Saunders S, Hammond JA, Carter D, Kirjavainen P, Anukam K, Reid G. Effect of bacterial vaginosis, Lactobacillus and Premarin estrogen replacement therapy on vaginal gene expression changes. Microbes Infect 2008;10:620-7.

68. Molenberghs G, Thijs H, Jansen I, Beunckens C, Kenward MG, Mallinckrodt C, Carroll RJ. Analyzing incomplete longitudinal clinical trial data. Biostatistics 2004;5:445-64.

69. Little RJA, Rubin DB. Statistical Analysis with Missing Data.New York: Wiley; 2002.

70. Carpenter JR, Kenward MG, White IR. Sensitivity analysis after multiple imputation under missing at random: a weighting approach. Stat Methods Med Res 2007;16:259-75.

71. Wood AM, White IR, Hillsdon M, Carpenter J. Comparison of imputation and modelling methods in the analysis of a physical activity trial with missing outcomes. Int J Epidemiol 2005;34:89-99.

72. Holmes I, Harris K, Quince C. Dirichlet multinomial mixtures: generative models for microbial metagenomics. PLoS One 2012;7:e30126. PMCID: PMC3272020

73. Lozupone CA, Hamady M, Kelley ST, Knight R. Quantitative and qualitative beta diversity measures lead to different insights into factors that structure microbial communities. Appl Environ Microbiol 2007;73:1576-85. PMCID: PMC1828774

74. Langille MG, Zaneveld J, Caporaso JG, McDonald D, Knights D, Reyes JA, Clemente JC, Burkepile DE, Vega Thurber RL, Knight R, Beiko RG, Huttenhower C. Predictive functional profiling of microbial communities using 16S rRNA marker gene sequences. Nat Biotechnol 2013;31:814-21. PMCID: PMC3819121

75. Upson K, Allison KH, Reed SD, Jordan CD, Newton KM, Swisher EM, Doherty JA, Garcia RL. Biomarkers of progestin therapy resistance and endometrial hyperplasia progression. Am J Obstet Gynecol 2012;207:36-8. PMCID: PMC3398620

76. Allison KH, Upson K, Reed SD, Jordan CD, Newton KM, Swisher EM, Garcia RL. PAX2 loss by immunohistochemistry occurs early and often in endometrial hyperplasia. Int J Gyn Pathol 2012;31:151-9.

77. Nilsson K, Heimer G. Low-dose oestradiol in the treatment of urogenital oestrogen deficiency--a pharmacokinetic and pharmacodynamic study. Maturitas 1992;15:121-7.

78. Notelovitz M, Funk S, Nanavati N, Mazzeo M. Estradiol absorption from vaginal tablets in postmenopausal women. Obstet Gynecol 2002;99:556-62.

79. Santen RJ, Pinkerton JV, Conaway M, Ropka M, Wisniewski L, Demers L, Klein KO. Treatment of urogenital atrophy with low-dose estradiol: preliminary results. Menopause 2002;9:179-87.

80. Ulrich LS, Naessen T, Elia D, Goldstein JA, Eugster-Hausmann M. Endometrial safety of ultra-low-dose Vagifem 10 microg in postmenopausal women with vaginal atrophy. Climacteric 2010;13:228-37.

81. Anderson GL, Limacher M, Assaf AR, Bassford T, Beresford SA, Black H, Bonds D, Brunner R, Brzyski R, Caan B, Chlebowski R, Curb D, Gass M, Hays J, Heiss G, Hendrix S, Howard BV, Hsia J, Hubbell A, Jackson R, Johnson KC, Judd H, Kotchen JM, Kuller L, LaCroix AZ, Lane D, Langer RD, Lasser N, Lewis CE, Manson J, Margolis K, Ockene J, O'Sullivan MJ, Phillips L, Prentice RL, Ritenbaugh C, Robbins J, Rossouw JE, Sarto G, Stefanick ML, Van HL, Wactawski-Wende J, Wallace R, Wassertheil-Smoller S. Effects of conjugated equine estrogen in postmenopausal women with hysterectomy: the Women's Health Initiative randomized controlled trial. JAMA 2004;291:1701-12.

82. Jick H, Derby LE, Myers MW, Vasilakis C, Newton KM. Risk of hospital admission for idiopathic venous thromboembolism among users of postmenopausal oestrogens. Lancet 1996;348:981-3.

83. Canonico M, Oger E, Plu-Bureau, Conard J, Meyer G, Levesque H, Trillot N, Barrellier MT, Wahl D, Emmerich J, Scarabin PY. Hormone therapy and venous thromboembolism among postmenopausal women: impact of the route of estrogen administration and progestogens: the ESTHER study. Circulation 2007;115:840-5.

84. Gomes MP, Deitcher SR. Risk of venous thromboembolic disease associated with hormonal contraceptives and hormone replacement therapy: a clinical review. Arch Intern Med 2004;164:1965-76.

85. Scarabin PY, Oger E, Plu-Bureau. Differential association of oral and transdermal oestrogen-replacement therapy with venous thromboembolism risk. Lancet 2003;362:428-32.

86. Renoux C, Dell'aniello S, Garbe E, Suissa S. Transdermal and oral hormone replacement therapy and the risk of stroke: a nested case-control study. BMJ 2010;340:c2519.

87. Grodstein F, Manson JE, Colditz GA, Willett WC, Speizer FE, Stampfer MJ. A prospective, observational study of postmenopausal hormone therapy and primary prevention of cardiovascular disease. Ann Intern Med 2000;133:933-41.

88. Bygdeman M, Swahn ML. Replens versus dienoestrol cream in the symptomatic treatment of vaginal atrophy in postmenopausal women. Maturitas 1996;23:259-63.
