# Supplementary material for: Association of Vaginal Estradiol Tablet With Serum Estrogen Levels in Women Who Are Postmenopausal: Secondary Analysis of a Randomized Clinical Trial
Source: JAMA Netw Open. 2022 Nov 14;5(11):e2241743. doi: 10.1001/jamanetworkopen.2022.41743 (PMC9664265; doi:10.1001/jamanetworkopen.2022.41743)
Supplement: Supplement 2. — eTable 1. Associations Between Baseline Participant Characteristics and Week 12 Estrone Concentration eTable 2. Associations Between Baseline Participant Characteristics and Week 12 SHBG Concentration eFigure 1. CONSORT Diagram of How Original Trial Participants Were Enrolled and How This Subset Was Selected eFigure 2. Serum Concentrations of Estrone at Enrollment and 12 Weeks for Participants in the MsFLASH Vaginal Health Randomized Trial Assigned to the Vaginal 10 μg Estradiol Tablet + Placebo Gel (n = 88) or Dual Placebo (n = 86) Arms eFigure 3. Serum Concentrations of Sex Hormone-Binding Globulin (SHBG) at Enrollment and 12 Weeks for Participants in the MsFLASH Vaginal Health Randomized Trial Assigned to the Vaginal 10 μg Estradiol Tablet + Placebo Gel (n = 88) or Dual Placebo (n = 86) Arms [file jamanetwopen-e2241743-s002.pdf]

Supplementary Online Content

Mitchell CM, Larson JC, Crandall CJ, et al. Association of vaginal estradiol tablet with serum estrogen levels in women who are postmenopausal: secondary analysis of a randomized clinical trial. *JAMA Netw Open*. 2022;5(11):e2241743. doi:10.1001/jamanetworkopen.2022.41743

eTable 1. Associations Between Baseline Participant Characteristics and Week 12 Estrone Concentration

eTable 2. Associations Between Baseline Participant Characteristics and Week 12 SHBG Concentration

eFigure 1. CONSORT Diagram of How Original Trial Participants Were Enrolled and How This Subset Was Selected

eFigure 2. Serum Concentrations of Estrone at Enrollment and 12 Weeks for Participants in the MsFLASH Vaginal Health Randomized Trial Assigned to the Vaginal 10 μg Estradiol Tablet + Placebo Gel (n = 88) or Dual Placebo (n = 86) Arms

eFigure 3. Serum Concentrations of Sex Hormone-Binding Globulin (SHBG) at Enrollment and 12 Weeks for Participants in the MsFLASH Vaginal Health Randomized Trial Assigned to the Vaginal 10 μg Estradiol Tablet + Placebo Gel (n = 88) or Dual Placebo (n = 86) Arms

This supplementary material has been provided by the authors to give readers additional information about their work.

© 2022 Mitchell CM et al. *JAMA Network Open.*

eTable 1. Associations between baseline participant characteristics and week 12 Estrone concentration

		**Week 12 Estrone (pg/mL)**:	
**Characteristic**	**N (%)**[a]	Geometric Mean (SD)	**p-value**[b]
Treatment group			0.78
Vaginal estrogen	88 (51)	11.8 (5.9)	
Placebo	86 (49)	11.5 (6.3)	
Study pill adherence, %			0.21
< 80	22 (13)	10.2 (4.7)	
≥ 80	152 (87)	11.9 (6.3)	
Age, years			0.29
< 60	72 (41)	12.3 (5.9)	
≥ 60	102 (59)	11.2 (6.2)	
Time since menopause, years			0.12
< 5	25 (14)	13.5 (7.5)	
≥ 5	147 (85)	11.3 (5.8)	
Body Mass Index, kg/m^2			0.001
< 25	82 (47)	10.2 (5.6)	
≥ 25	92 (53)	13.1 (6.1)	
Estradiol concentration			0.002
< median (3.5 pg/mL)	87 (50)	10.3 (5.3)	
≥ median	87 (50)	13.2 (6.6)	
Estrone concentration			<0.001
< median (10.5 pg/mL)	87 (50)	8.8 (4.1)	
≥ median	87 (50)	15.5 (6.3)	
SHBG concentration			0.27
< median (59.3 nmol/L)	87 (50)	12.2 (5.9)	
≥ median	87 (50)	11.2 (6.2)	
Race			0.01
African American	8 (5)	20.0 (8.5)	
Other or unknown	14 (8)	10.9 (5.0)	
White	152 (87)	11.4 (5.9)	
Sexually Active			0.79
Yes	143 (82)	11.7 (6.0)	
No	30 (17)	11.4 (6.4)	
Most Bothersome Symptom			0.26
Pain with vaginal penetration	97 (56)	11.3 (5.4)	
Vaginal dryness	38 (22)	11.5 (6.8)	
Vulvar / vaginal itching	15 (9)	15.2 (6.3)	
Vulvar / vaginal irritation	13 (8)	11.7 (6.6)	
Vulvar / vaginal soreness	7 (4)	14.3 (8.1)	
pH			0.20
≤ 5	22 (13)	13.3 (6.6)	
> 5	151 (87)	11.4 (6.0)	
Vaginal Maturation Index, % superficial cells			0.08
≤ 5	143 (82)	11.5 (6.1)	
> 5	11 (6)	15.4 (8.9)	

© 2022 Mitchell CM et al. *JAMA Network Open.*

MENQOL total			0.27
< median (< 3.16)	83 (48)	11.1 (5.3)	
≥ median	84 (48)	12.1 (6.9)	
PHQ-8 depression			0.27
No symptoms (< 5)	121 (70)	11.3 (5.4)	
Mild or worse symptoms	52 (30)	12.5 (7.7)	
GAD-7 anxiety			0.30
No symptoms (< 5)	108 (62)	11.3 (5.6)	
Mild or worse symptoms	65 (37)	12.3 (7.0)	
ISI insomnia			0.18
No symptoms (< 8)	92 (53)	11.1 (5.2)	
Mild or worse symptoms	81 (47)	12.4 (7.1)	

[a]Percentages may not add up to 100% due to missing characteristic data
[b]p-value from an ANOVA model of log-transformed week 12 Estrone by category of participant characteristic

e**Table 2**. Associations between baseline participant characteristics and week 12 SHBG concentration

		**Week 12 SHBG (nmol/L)**:	
**Characteristic**	**N (%)**[a]	Geometric Mean (SD)	**p-value**[b]
Treatment group			0.48
Vaginal estrogen	88 (51)	56.7 (26.8)	
Placebo	86 (49)	52.9 (42.1)	
Study pill adherence, %			0.93
< 80	22 (13)	54.2 (36.7)	
≥ 80	152 (87)	54.9 (35.7)	
Age, years			0.03
< 60	72 (41)	62.4 (25.8)	
≥ 60	102 (59)	50.0 (38.3)	
Time since menopause, years			0.41
< 5	25 (14)	60.4 (19.1)	
≥ 5	147 (85)	53.7 (37.3)	
Body Mass Index, kg/m^2			<0.001
< 25	82 (47)	68.5 (25.1)	
≥ 25	92 (53)	45.0 (34.9)	
Estradiol concentration			0.18
< median (3.5 pg/mL)	87 (50)	58.5 (45.4)	
≥ median	87 (50)	51.3 (25.4)	
Estrone concentration			0.72
< median (10.5 pg/mL)	87 (50)	55.8 (44.9)	
≥ median	87 (50)	53.8 (24.5)	
SHBG concentration			<0.001
< median (59.3 nmol/L)	87 (50)	37.8 (26.9)	
≥ median	87 (50)	79.5 (20.7)	
Race			0.83
African American	8 (5)	62.2 (27.3)	
Other or unknown	14 (8)	56.7 (18.5)	
White	152 (87)	54.3 (37.1)	
Sexually Active			0.36
Yes	143 (82)	55.9 (25.8)	
No	30 (17)	49.6 (60.4)	
Most Bothersome Symptom			0.33
Pain with vaginal penetration	97 (56)	55.1 (24.0)	
Vaginal dryness	38 (22)	59.9 (26.4)	
Vulvar / vaginal itching	15 (9)	49.1 (28.9)	
Vulvar / vaginal irritation	13 (8)	39.6 (71.7)	
Vulvar / vaginal soreness	7 (4)	63.3 (27.3)	
pH			0.91
≤ 5	22 (13)	55.5 (27.6)	
> 5	151 (87)	54.6 (36.8)	
Vaginal Maturation Index, % superficial cells			0.65
≤ 5	143 (82)	54.0 (37.3)	
> 5	11 (6)	49.0 (26.9)	

© 2022 Mitchell CM et al. *JAMA Network Open.*

MENQOL total			0.18
< median (< 3.16)	83 (48)	51.6 (41.6)	
≥ median	84 (48)	59.0 (25.4)	
PHQ-8 depression			0.85
No symptoms (< 5)	121 (70)	55.1 (26.1)	
Mild or worse symptoms	52 (30)	54.0 (51.6)	
GAD-7 anxiety			0.87
No symptoms (< 5)	108 (62)	55.1 (27.1)	
Mild or worse symptoms	65 (37)	54.2 (46.8)	
ISI insomnia			0.82
No symptoms (< 8)	92 (53)	55.4 (26.6)	
Mild or worse symptoms	81 (47)	54.1 (43.8)	

[a]Percentages may not add up to 100% due to missing characteristic data

[b]p-value from an ANOVA model of log-transformed week 12 SHBG by category of participant characteristic

© 2022 Mitchell CM et al. *JAMA Network Open.*

eFigure 1: CONSORT diagram of how original trial participants were enrolled and how this subset was selected.

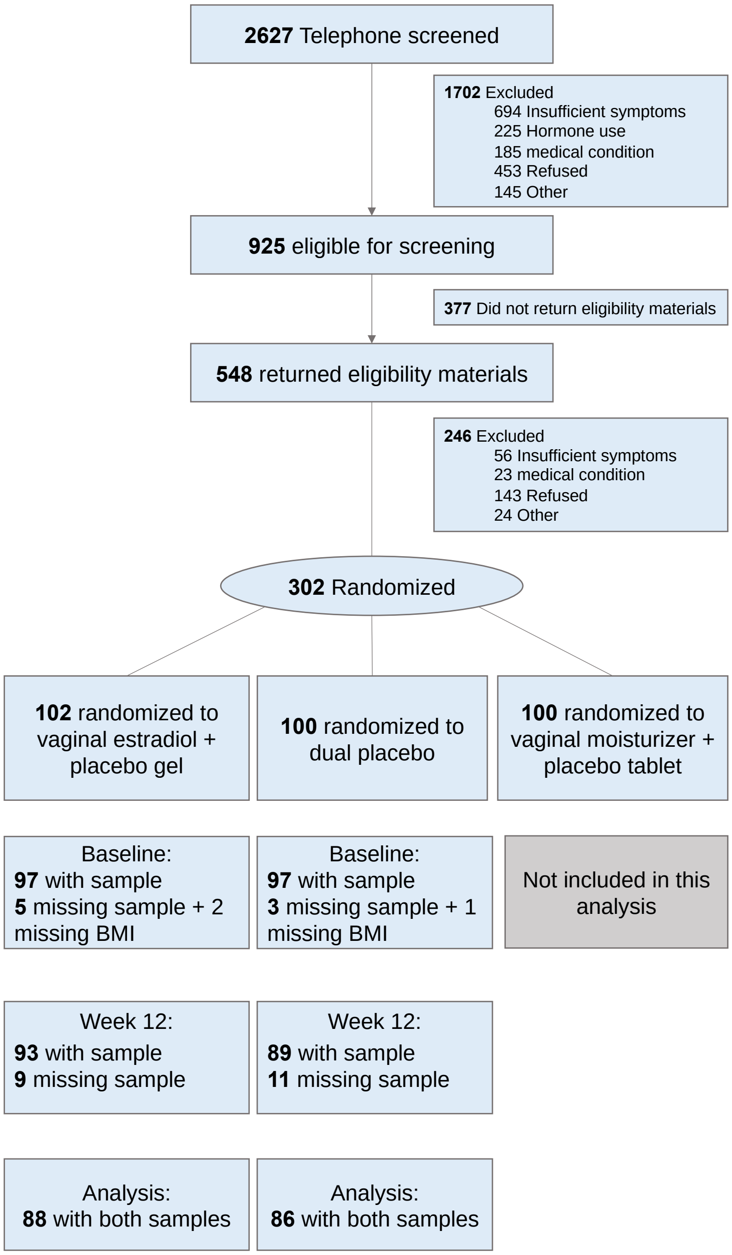

© 2022 Mitchell CM et al. *JAMA Network Open.*

eFigure 2: Serum concentrations of estrone at enrollment and 12 weeks for participants in the MsFLASH Vaginal Health randomized trial assigned to the vaginal 10 µg estradiol tablet + placebo gel (n = 88) or dual placebo (n = 86) arms. Each triangle represents a measurement from an individual participant, and the lines connect the pre-treatment and week 12 values for each participant.

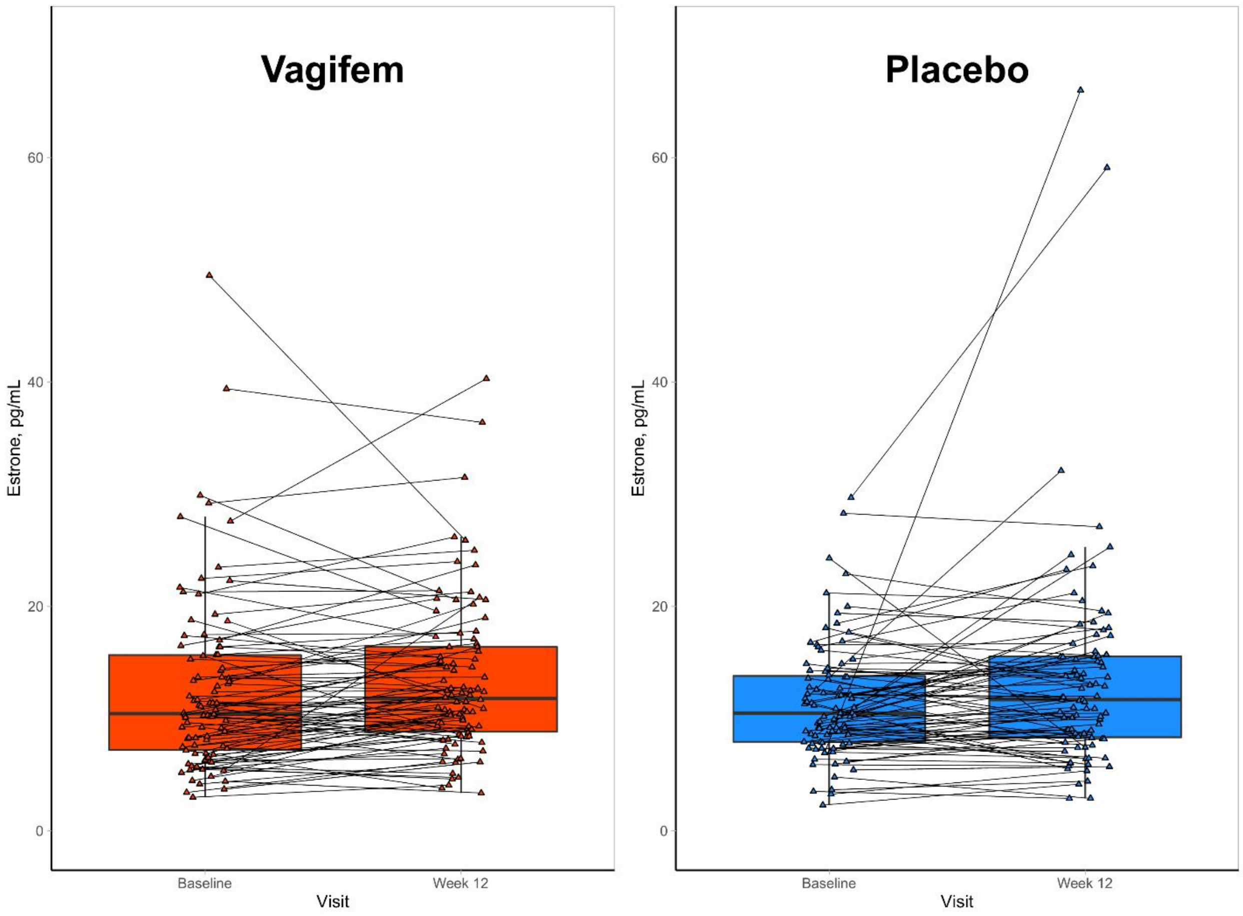

© 2022 Mitchell CM et al. *JAMA Network Open.*

eFigure 3: Serum concentrations of sex hormone binding globulin (SHBG) at enrollment and 12 weeks for participants in the MsFLASH Vaginal Health randomized trial assigned to the vaginal 10 µg estradiol tablet + placebo gel (n = 88) or dual placebo (n = 86) arms. Each triangle represents a measurement from an individual participant, and the lines connect the pre-treatment and week 12 values for each participant.

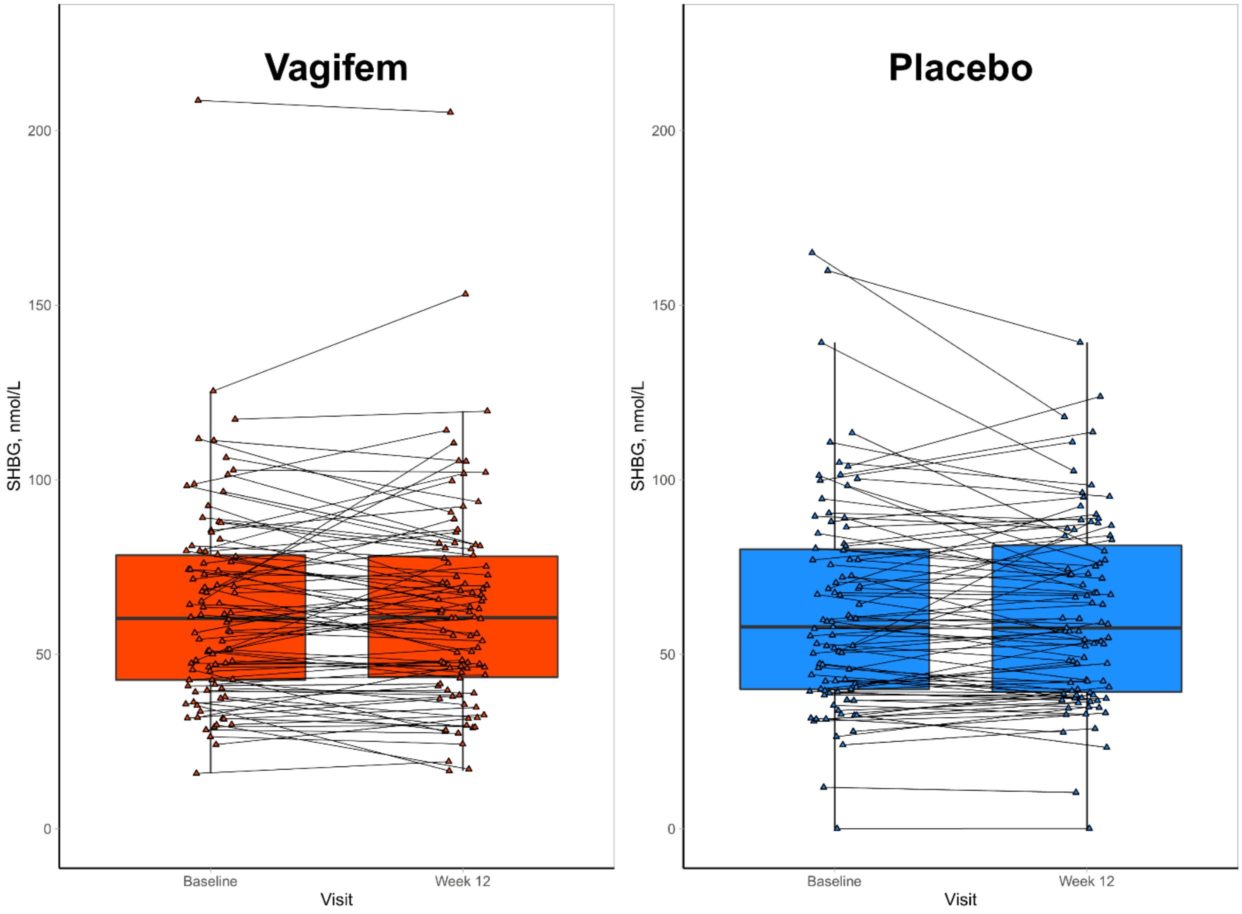

© 2022 Mitchell CM et al. *JAMA Network Open.*
