# Supplementary material for: Association of Vaginal Estradiol Tablet With Serum Estrogen Levels in Women Who Are Postmenopausal: Secondary Analysis of a Randomized Clinical Trial
Source: JAMA Netw Open. 2022 Nov 14;5(11):e2241743. doi: 10.1001/jamanetworkopen.2022.41743 (PMC9664265; doi:10.1001/jamanetworkopen.2022.41743)
Supplement: Supplement 3. — Data Sharing Statement [file jamanetwopen-e2241743-s003.pdf]

Data Sharing Statement

Mitchell. Association of Vaginal Estradiol Tablet With Serum Estrogen Levels in Women Who Are Postmenopausal. *JAMA Netw Open*. Published November 14, 2022. doi:10.1001/jamanetworkopen.2022.41743

Data
Data available: Yes
Data types: Deidentified participant data, Data dictionary
How to access data: The MsFLASH Data Coordinating Center (DCC) has a National Institute of Aging (NIA)-approved Data Sharing Plan. The DCC has datasets prepared with all data elements with certain deletions and recoding to protect study participant confidentiality. These datasets include documentation in electronic form. Dr. Guthrie, the study PI, is the primary contact: kguthrie@fredhutch.org. Once MsFLASH funding has expired, datasets will be supplied to the NIA and applications will be reviewed by the NIA Project Scientist.
When available: With publication

Supporting Documents
Document types: None

Additional Information
Who can access the data: Researchers whose proposed use of the data has been approved
Types of analyses: Any purpose
Mechanisms of data availability: An outside investigator requesting study data will complete a manuscript proposal, providing the specific research question being considered, an overview of the methodology to be applied, and academic titles/mentors of the investigators. The MsFLASH PIs will review these proposals and accept those from qualified investigators. Clearly frivolous requests from unqualified investigators will be screened out. The proposal will then be sent to Network investigators for enlistment of a study sponsor, that is, an investigator with knowledge of the MsFLASH studies that can serve as a co-author. As outlined in the NIH Data Sharing Policy, investigators must sign a Data Use Agreement before obtaining access to datasets.
Any additional restrictions: The MsFLASH grant is close to expiration, at which time the data will be transferred to NIA, which may have a different set of requirements for their access.
